# Dynamics of release factor recycling during translation termination in bacteria

**DOI:** 10.1093/nar/gkad286

**Published:** 2023-04-27

**Authors:** Arjun Prabhakar, Michael Y Pavlov, Jingji Zhang, Gabriele Indrisiunaite, Jinfan Wang, Michael R Lawson, Måns Ehrenberg, Joseph D Puglisi

**Affiliations:** Department of Structural Biology, Stanford University School of Medicine, Stanford, CA 94305-5126, USA; Program in Biophysics, Stanford University, Stanford, CA 94305-5126, USA; Department of Cell and Molecular Biology, Biomedical Center, Box 596, Uppsala University, Uppsala, Sweden; Department of Structural Biology, Stanford University School of Medicine, Stanford, CA 94305-5126, USA; Department of Cell and Molecular Biology, Biomedical Center, Box 596, Uppsala University, Uppsala, Sweden; Department of Structural Biology, Stanford University School of Medicine, Stanford, CA 94305-5126, USA; Department of Structural Biology, Stanford University School of Medicine, Stanford, CA 94305-5126, USA; Department of Cell and Molecular Biology, Biomedical Center, Box 596, Uppsala University, Uppsala, Sweden; Department of Structural Biology, Stanford University School of Medicine, Stanford, CA 94305-5126, USA

## Abstract

In bacteria, release of newly synthesized proteins from ribosomes during translation termination is catalyzed by class-I release factors (RFs) RF1 or RF2, reading UAA and UAG or UAA and UGA codons, respectively. Class-I RFs are recycled from the post-termination ribosome by a class-II RF, the GTPase RF3, which accelerates ribosome intersubunit rotation and class-I RF dissociation. How conformational states of the ribosome are coupled to the binding and dissociation of the RFs remains unclear and the importance of ribosome-catalyzed guanine nucleotide exchange on RF3 for RF3 recycling *in vivo* has been disputed. Here, we profile these molecular events using a single-molecule fluorescence assay to clarify the timings of RF3 binding and ribosome intersubunit rotation that trigger class-I RF dissociation, GTP hydrolysis, and RF3 dissociation. These findings in conjunction with quantitative modeling of intracellular termination flows reveal rapid ribosome-dependent guanine nucleotide exchange to be crucial for RF3 action *in vivo*.

## Graphical Abstract

**Graphical Abstract:**
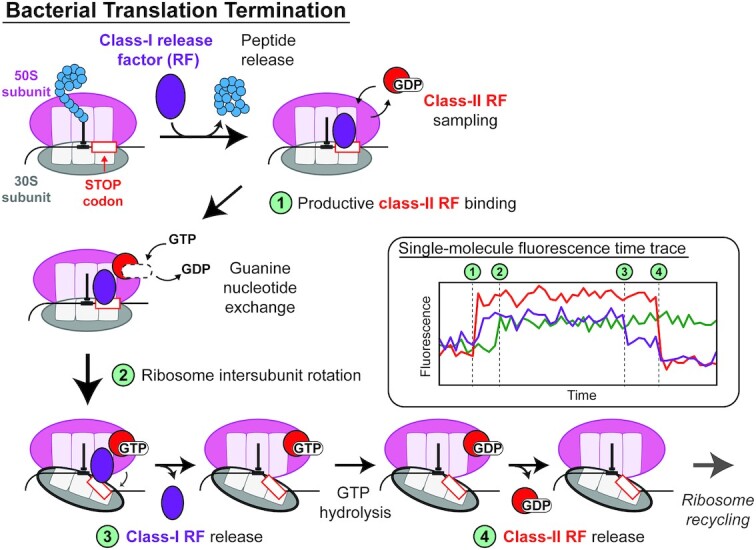
Single-molecule tracking of translation termination reveals that class-II release factor (RF) first samples the post-termination ribosome. Then, after a productive binding event, class-II RF in GTP form catalyzes ribosome rotation causing class-I RF dissociation and GTP hydrolysis that leads to rapid class-II RF dissociation in GDP form. Kinetic modeling highlights crucial importance of ribosome-accelerated GDP to GTP exchange on class-II RF for its catalytic action in growing cells.

## INTRODUCTION

Termination of cellular protein synthesis begins when an mRNA stop codon is translocated into the aminoacyl-tRNA binding site (A site) of the ribosome. This triggers A-site binding of a class-I release factor (RF), which promotes hydrolytic release of the nascent polypeptide chain (1–3). In bacteria, class-I RFs RF1 and RF2 decode stop codons UAG or UAA and UGA or UAA, respectively. Stop-codon recognition leads to a conformational change of the class-I RF, which brings its GGQ motif (4) into the ribosomal peptidyl transferase center (PTC) (5) to promote rapid hydrolysis of the ester bond linking the nascent peptide chain to the tRNA at the peptidyl-tRNA binding site (P site) (6–12).

*In vivo* concentrations of RF1 and RF2 are sub-stoichiometric to ribosome concentration (13), meaning that rapid class-I RF cycling may be important for hastening termination in the living cell (14). Dissociation of class-I RFs from the post-termination ribosome (15,16) is accelerated by a class-II RF, the GTPase RF3, in a GTP -dependent manner (14). RF3 action also minimizes the inhibitory effect of post-termination class-I RF rebinding on ribosomal recycling by RRF and EF-G (15,17,18). RF3 deficiency decreases growth rate, increases stop-codon readthrough frequency and induces cold sensitivity of *Escherichia coli* cells (19,20). These phenotypes may arise from lower termination efficiency caused by slower recycling of RF1 and RF2 or lack of sense error correction by RF3-dependent post-peptidyl transfer control (21). Bacterial sensitivity to class-I RF dissociation rate reduction makes this step a viable target of antimicrobial agents, such as Apidaecin 137 (Api137), which stabilize RF1 on the post-termination ribosome (22).

The time for exchange of GDP to GTP on RF3 free in solution is 30s and is dominated by GDP dissociation from RF3 (23). The exchange is much faster for RF3 in complex with the class-I RF-bound post-termination ribosome (16,23), making the ribosome a nucleotide exchange factor for RF3. From the lower binding affinity of GTP than GDP to RF3 in solution and the requirement for rapid RF3 recycling in bacterial termination, it was suggested that in the living cell it is RF3·GDP that normally enters the pre-termination ribosome. Then GDP dissociates rapidly from RF3 which allows for rapid binding of solution-phase GTP into ribosome-bound apo-RF3 (12,23).

The proposition that the ribosome is a guanine nucleotide exchange factor for RF3 has been confirmed (16,24–26). However, fluorescence experiments estimated just a four-fold higher affinity of RF3 to GDP than GTP, which, in conjunction with the estimated [GTP]/[GDP] ratio of 10 *in vivo* (27) and the assumption that RF3·GDP and RF3·GTP are equilibrated off the ribosome *in vivo*, led to the proposal that in the cell cytoplasm RF3 must be mainly in the GTP form and that ribosome-catalyzed guanine nucleotide exchange is redundant for RF3 function (16).

Structural and dynamic data suggest that the mechanism of class-I RF recycling between free and ribosome-bound states involves changes in the global ribosome conformation. The 70S ribosome has two global conformations that differ by counterclockwise rotation of 6–12 degrees of the small with respect to the large subunit, here called the non-rotated and rotated ribosomal states, respectively. Termination complexes bound to either RF1 or RF2 were observed in the non-rotated state, whereas complexes bound to RF3·GDPNP were observed in the rotated state (24,28), consistent with the hypothesis that RF3-induced intersubunit rotation accelerates the dissociation of class-I RFs, as further supported by cryo-EM structures of ribosomal termination complex with RF1 and RF3 (29). Thus, there is consensus regarding the importance of intersubunit rotation during termination, but its timing in relation to release factor binding and dissociation events and GTP hydrolysis on RF3 remains disputed. From Förster Resonance Energy Transfer (FRET) studies of RF1 and RF3 dissociation kinetics it was proposed that GTP hydrolysis accelerates sequential dissociation of first RF3 and then RF1 (30). From another study using single-molecule FRET (smFRET) it was suggested that GTP hydrolysis is not required for either RF1/RF2 or RF3 dissociation and that the order RF1/RF2 and RF3 dissociation events is stochastic rather than sequential (24).

To resolve these ambiguities regarding the interplay between nucleotide state and ribosomal rotation in the RF3 mechanism, we applied here bulk and real-time single-molecule kinetics to determine the timings of 70S rotation, class-I RF and class-II RF dissociation events during translation termination. We then used quantitative modeling of protein synthesis in growing *E. coli* cells to answer the question if ribosome-dependent acceleration of guanine nucleotide exchange on RF3 (16,23) is crucial for RF3 action. The combined approach of detailed kinetics experiments with global termination modeling have provided novel insights in the termination mechanism of bacteria. Our findings highlight its dynamic nature and the importance of using the cellular context to assess the functional meaning of *in vitro* data.

## MATERIALS AND METHODS

### Single-molecule assays

#### Reagents and buffers for single-molecule experiment

*Escherichia coli* ribosomal subunits used in all single-molecule experiments were purified as described before (31). To label the ribosomal subunits specifically with fluorescent dyes, hairpin loop extensions were introduced into phylogenetically variable, surface-accessible loops of the *E. coli* 16S rRNA in helix 44 and 23S rRNA in helix 101 using previously described site-directed mutagenesis (32). The 70S ribosomes were purified from SQ380 cells expressing these mutant ribosomes, and the 30S and 50S subunits were prepared from dissociated 70S particles using previously described protocols (31).

IF2, EF-Tu, EF-G, EF-Ts, RRF and ribosomal protein S1 from *E. coli* were purified from overexpressing strains as previously described (18,31). fMet-tRNA^fMet^, Lys-tRNA^Lys^ and Phe-tRNA^Phe^ were charged and purified according to published protocols (33,34). The mRNAs used, MF-UAA and 6FK-UAA, contains a 5′-biotin followed by a 5′-UTR and Shine-Dalgarno sequence derived from gene 32 of the T4 phage upstream of the AUG start codon. For both mRNAs, there are four spacer Phe codons downstream of the UAA stop codons. Both mRNAs were chemically synthesized by Dharmacon.

Cy5.5-labeled RF1 and RF2 were generated from single-cysteine variants of the proteins which were purified with previously described protocol (18). The purified proteins were incubated with 30-fold excess of the Cy5.5-maleimide dye (Lumiprobe) for 24 h at 4°C. Removal of free dye and storage was done as previously described (18).

Wild-type RF3 was purified by overexpressing them in *E. coli* BL21(DE3) cells transformed with pET-His6-MBP-Asn10-TEV-LIC cloning vector (QB3‐Berkeley Macrolab) containing an N-terminal six-histidine (6xHis) affinity tag, a maltose binding protein (MBP), linker of ten asparagines (N10), TEV protease cleavage site, followed by the RF3 gene from *E. coli* MG1655 K12 strain. Cells were lysed using sonication, and the lysate clarified by centrifugation was loaded onto a 5-ml HiTrap Ni^2+^ column (GE Healthcare). The fractions containing the protein were dialyzed in the presence of TEV protease to cleave the N-terminal 6xHis-MBP-N10 tag. After flowing this cleaved protein through a column containing 1 ml Ni-NTA Agarose resin (Qiagen), the protein was then purified on a size-exclusion column (Superdex 200 26/60, GE Healthcare).

Cy5-RF3 was generated by enzymatically functionalizing Cy5-labeled Coenzyme A (CoA) on RF3 with an N-terminal ybbR peptide tag (DSLEFIASKLA) using Sfp synthase. Sfp synthase was purified using previously described protocol (35). Cy5-labeled CoA was generated using previously described protocol (36). ybbR-tagged RF3 was purified similarly to the wild-type RF3 with the insertion of the ybbR sequence upstream of the RF3 gene in pET-His6-MBP-Asn10-TEV-LIC vector. Purified ybbR-RF3 was incubated with 6-fold excess of Cy5-CoA and 5:4 molar ratio to Sfp synthase at 37°C for 1 h in buffer containing 50 mM HEPES–KOH, pH 7.5, 100 mM NaCl, 10 mM MgCl_2_ and 1 mM DTT. After the reaction, the mixture was passed through a column containing 1 ml Ni-NTA Agarose resin (Qiagen) to remove Sfp synthase and two 10DG desalting gravity columns (Bio-Rad) to remove free dye. This sample was then finally loaded onto a size-exclusion column (Superdex 200 10/300 GL, GE Healthcare) to remove any residual free dye/enzyme. The labeled RF3 was stored in storage buffer (20 mM Tris–HCl pH 7.5 at 25°C, 100 mM KCl, 2.5 mM MgCl_2_, 1 mM DTT, 50% w/w glycerol) at −20°C.

All single-molecule experiments were conducted in a Tris-based polymix buffer consisting of 50 mM Tris-acetate (pH 7.5), 100 mM potassium chloride, 5 mM ammonium acetate, 0.5 mM calcium acetate, 5 mM magnesium acetate, 0.5 mM EDTA, 5 mM putrescine–HCl and 1 mM spermidine. Prior to the single-molecule experiments, the purified 30S and 50S ribosomal subunits (final concentration 1 μM) were mixed in 1:1 ratio with the dye-labeled DNA oligonucleotides complementary to the mutant ribosome hairpin extensions (31,32) at 37°C for 10 min and then at 30°C for 20 min in the Tris-based polymix buffer system. The 30S subunit was labeled with 5’-Cy3B-labeled DNA and the 50S subunit was labeled with 3’-BHQ-2-labeled DNA.

#### Single-molecule intersubunit FRET/factor binding experiments

The 30S pre-initiation complexes (PICs) were formed as described by incubating the following at 37°C for 5 min: 0.25 μM Cy3B-30S, pre-incubated with stoichiometric S1, 1 μM IF2, 1 μM fMet-tRNA^fMet^, 1 μM mRNA, and 4 mM GTP to form 30S PICs in the polymix buffer (37). Before use, we pre-incubate a SMRT Cell v3 from Pacific Biosciences (Menlo Park, CA, USA), a zero-mode waveguide (ZMW) chip, with 0.2% w/w Tween 20 in 50 mM Tris-acetate pH 7.5 and 50 mM KCl at room temperature for 10 min. After washing the chip in cell-washing buffer (50 mM Tris-acetate pH 7.5, 100 mM potassium chloride, 5 mM ammonium acetate, 0.5 mM calcium acetate, 5 mM magnesium acetate and 0.5 mM EDTA), the chip was incubated with a 1 mg/ml Neutravidin solution in 50 mM Tris-acetate pH 7.5 and 50 mM KCl at room temperature for 5 min. The cell was then washed with cell-washing buffer again. The formed 30S PICs were diluted with our Tris- based polymix buffer containing 4 mM GTP down to 10 nM PIC concentration. The diluted PICs were then loaded into the SMRT cell at room temperature for 3 min to immobilize the 30S PICs into the ZMW wells. Any excessive unbound material was washed away with our Tris-based polymix buffer containing 4 mM GTP. The immobilized 30S PICs were immersed with 20 μl of our Tris-based polymix buffer containing 4 mM GTP, 5 mg/mL BSA, 1 μM blocking dsDNA oligonucleotide, 2.5 mM Trolox, and a PCA/PCD oxygen scavenging system (2.5 mM 3,4-dihydroxybenzoic acid and 250 nM protocatechuate deoxygenase (38).

Ternary complexes (TCs) were formed between charged Phe-tRNA^Phe^ or Lys-tRNA^Lys^*E. coli* tRNAs and EF-Tu(GTP) by incubating in bulk the tRNA, EF-Tu, EF-Ts, GTP and energy regeneration system (phosphoenolpyruvate and pyruvate kinase). The TCs were added to the delivery mix at final concentration of 200 nM along with 200 nM BHQ-2–50S, 200 nM EF-G, 10–20 nM Cy5.5-RF1 or RF2, 0–400 nM Cy5- or dark RF3, 0–40 μM RRF, 4 mM GTP or GDPCP, 5 mg/ml BSA, 1 μM blocking dsDNA oligo, 2.5 mM Trolox, and the oxygen scavenging system (PCA and PCD) in polymix buffer.

**Figure 1.**
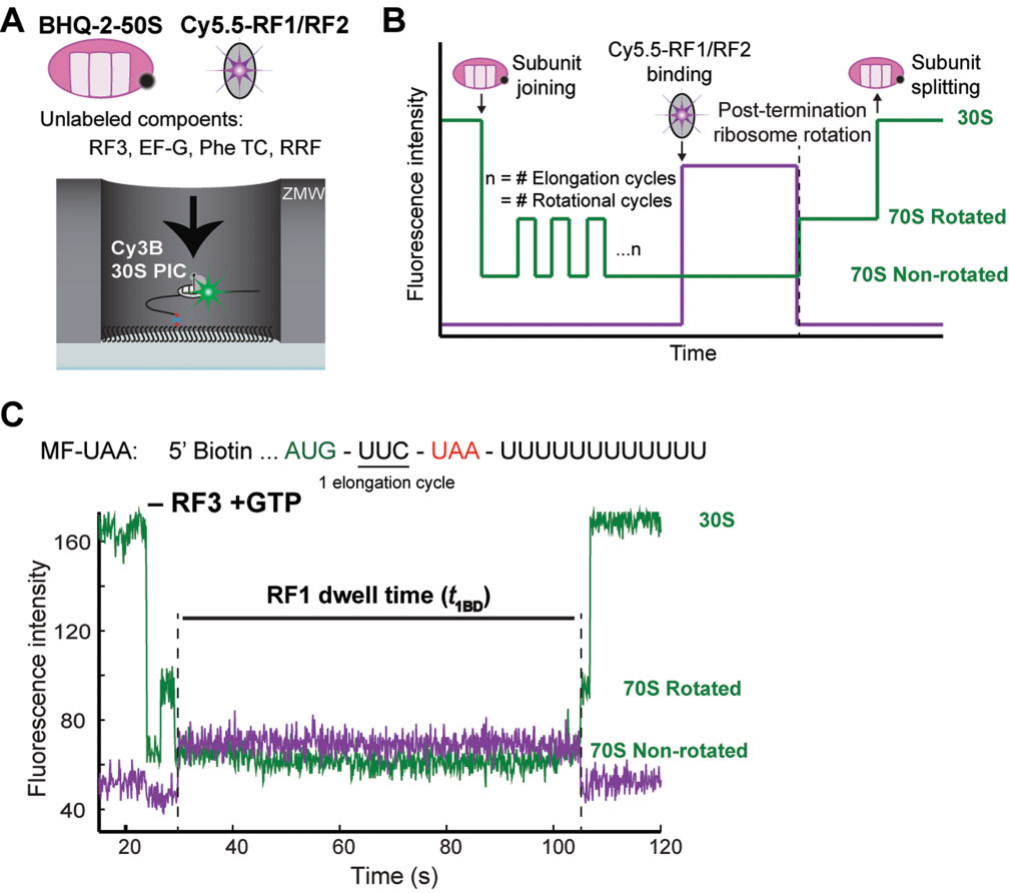
Real-time single-molecule translation assay setup. (**A**) In all experiments, 30S preinitiation complexes (PIC) containing Cy3B-30S, fMet-tRNA^fMet^ and IF2-GTP is immobilized on the surface of the ZMW wells via biotinylated mRNAs. The reaction is started by delivery of BHQ-2–50S, aa-tRNA, EF-Tu, EF-G, RRF, GTP and Cy5.5-labeled RF1 or RF2 and RF3. (**B**) Expected sequence of fluorescence signals from Cy3B (green) tracking ribosome conformation and Cy5.5 (purple) tracking binding of RF1 or RF2. Upon delivery of reagents, we observe 50S subunit joining (quenching of Cy3B signal), followed by cycles of ribosome rotations between the non-rotated and rotated states that represent the number of elongation cycles before the stop codon is translocated into the ribosomal A site. This is followed by RF1/RF2 binding (Cy5.5 signal increase), post-termination ribosome rotation, and RF1/RF2 dissociation (Cy5.5 signal decrease). The rotated post-termination ribosomes undergo subunit splitting (de-quenching of Cy3B signal) catalyzed by RRF and EF-G. (**C**) Top: codon sequence of the MF-UAA mRNA construct. Bottom: representative trace of Cy3B- and BHQ-2-labeled ribosome translating MF-UAA mRNA in presence of Phe-tRNA, EF-Tu,GTP, EF-G, RRF and Cy5.5-RF1 with Cy5.5-RF1 stochastic dwell time (${t}_{1BD}$) defined as the time interval between binding and dissociation of Cy5.5-RF1.

Before starting an experiment, the SMRT Cell was loaded into a modified PacBio RSII sequencer. At the start of the experiment, the instrument illuminates the SMRT cell with a green laser and then transfers 20 μl of a delivery mixture onto the cell surface at *t* = 10 s (see schematics in Figure 1A). All experiments were performed at either 20°C or 30°C, and data were collected for 6 min. Since the SMRT Cell contains 30S PICs immersed in 20 μl of polymix mixture, the final concentration of all the components during the experiment was 100 nM BHQ-2–50S, 100 nM TC, 100 nM EF-G, 0–50 nM Cy5.5-RF1 or RF2, 0–200 nM Cy5- or dark RF3, 0–20 μM RRF, 4 mM GTP or GDPCP, 5 mg/ml BSA, 1 μM blocking dsDNA oligo, 2.5 mM Trolox, 2.5 mM PCA and 250 nM PCD.

#### Cy5-RF3·GDPCP and dark RF3·GDPCP and cy5-RF3·GDP experiments on pre-elongated 70S ribosomes

For experiments involving Cy5-RF3·GDPCP, dark RF3·GDPCP or Cy5-RF3·GDP, reagents were delivered to pre-elongated 70S ribosomes with the stop codon in the A site. To form the immobilized 70S complex, we first immobilized Cy3B-30S Pre-Initiation Complex (PIC) on the ZMW chip as described above. After immobilization and wash, a reaction mixture containing the 50S subunit and elongation components (200 nM BHQ-2–50S, 200 nM TC, 200 nM EF-G and 4 mM GTP in Tris-based polymix buffer) was manually delivered to the chip and incubated for 2 min. Afterwards, the chip surface was rinsed with polymix buffer containing 4 mM GDPCP/GDP to wash off GTP and unbound factors. Samples were then imaged for 8 min using PacBio RSII instrument. For the Cy5-RF3·GDP experiments, 75 nM Cy5-RF3, 20 nM Cy5.5-RF1 and 4 mM GDP with oxygen scavenging factors in polymix buffer were delivered at the start of imaging. For the Cy5-RF3·GDPCP experiments, 75 nM Cy5-RF3, 20 nM Cy5.5-RF1 and 4 mM GDPCP with oxygen scavenging factors in polymix buffer were delivered at the start of imaging. For the dark RF3·GDPCP experiments, 0–75 nM dark RF3, 20 nM Cy5.5-RF1 and 4 mM GDPCP with oxygen scavenging factors in polymix buffer were delivered at the start of imaging.

#### PacBio RSII instrumentation and data analysis

Single-molecule intersubunit FRET and factor occupancy experiments were conducted using a commercial PacBio RSII sequencer that was modified to allow the collection of single-molecule fluorescence intensities from individual zero-mode waveguide (ZMW) wells about 130 nm in diameter in four different dye channels corresponding to Cy3, Cy3.5, Cy5 and Cy5.5 fluorescence (39). The RSII sequencer has two lasers for dye excitation at 532 nm and 632 nm. In all the Cy5.5-RF1 and Cy5.5-RF2 experiments, data was collected at 10 frames/s (100 ms exposure time) for 6 min using energy flux settings of the green laser at 0.72 mW/mm^2^ and red laser at 0.10 mW/mm^2^.

Data analyses for all experiments were conducted with MATLAB (MathWorks) scripts written in-house (39). ZMW wells containing fluorescently-labeled ribosomes were initially selected via an automated process based on fluorescence intensity, fluorescence lifetime and the changes in intensity. ZMWs with single productive ribosome complexes were then manually selected based on the expected changes in Cy3B fluorescence signal due to ribosome conformational changes and in Cy5 and Cy5.5 fluorescence signal due to RF occupancy changes. For most of the experiments, unless otherwise noted, productive complex was detected by the sequential changes in Cy3B and Cy5.5 fluorescence as shown in Figure 1B. Productive complex in the pre-elongated ribosome experiments described in the previous section is defined by the burst of Cy5.5 fluorescence signal due to RF1 binding. All conformational and compositional states defined by fluorescence were assigned as previously described (39) based on a hidden Markov model based approach and visually corrected. All lifetimes were plotted as cumulative distributions and fitted to single-, double-, or triple-exponential functions using curve-fitting tool on MATLAB.

#### Determination of rate constants from single-molecule data

Unless specified otherwise, all rate constants (*k*) were calculated by fitting the cumulative distribution of the corresponding dwell times as functions of time (*t*). The cumulative distribution corresponds to the probability that an event has occurred between times 0 and *t*, where the probability goes to one when *t* goes to infinity. In the simplest case where dissociation is determined by a single rate constant the cumulative distribution *f*(*t*) is a single exponential:


(1) $$\begin{equation*}{{f(t) = a}} \cdot {\rm{(1 - }}{{\rm{e}}}^{{{ - kt}}}{\rm{),}}\,{{a = 1}}\end{equation*}$$

In the presence of only GTP, GDP or GDPCP the cumulative distributions of RF3 occupancy times on the non-rotated ribosome were all biphasic, and fitted to double exponential functions:


(2) $$\begin{equation*}{{f(t) = }}{{{a}}}_1{\rm{(1 - }}{{\rm{e}}}^{{{ - }}{{{k}}}_1{\rm{t}}}{\rm{) + }}{{{a}}}_2{\rm{(1 - }}{{\rm{e}}}^{{\rm{ - }}{{{k}}}_2{{t}}}{\rm{)}}\,,\,{{{a}}}_{\rm{1}}{\rm{ + }}{{{a}}}_{{2}}{\rm{ = 1}}\end{equation*}$$

where *a*_1_ and *a*_2_ are the amplitudes of contribution of the two rate constants *k*_1_ and *k*_2_. In the presence of 50% GTP and 50% GDP the cumulative distributions of RF3 occupancy times on the non-rotated ribosome were fitted to triple-exponential functions:


(3) $$\begin{eqnarray*}{{f = }}{{{a}}}_1{\rm{(1 - }}{{\rm{e}}}^{{\rm{ - }}{{{k}}}_1{{t}}}{\rm{) + }}{{{a}}}_2{\rm{(1 - }}{{\rm{e}}}^{{\rm{ - }}{{{k}}}_2{{t}}}{\rm{) + }}{{{a}}}_3{\rm{(1 - }}{{\rm{e}}}^{{\rm{ - }}{{{k}}}_3{{t}}}{\rm{),}}\,{{{a}}}_{\rm{1}}{\rm{ + }}{{{a}}}_{\rm{2}}{\rm{ + }}{{{a}}}_{\rm{3}}{\rm{ = 1}}\nonumber\\ \end{eqnarray*}$$

where *a*_1_, *a*_2_ and *a*_3_ are the amplitudes of contribution of the rate constants *k*_1_, *k*_2_ and *k*_3_.

We model the RF3 concentration, [RF3], dependence of mean class-I RF (class-I RF1 and RF2 are denoted here as RF_i_, where i = 1 or 2) dwell time${\tau }_{iBD}$${\tau }_{iBD}$, defined as the mean time interval from RF_i_ binding to the ribosome till RF_i_ dissociation from it, with two kinetic schemes, one for first-time RF_i_ bound (‘first-bound’) and the other for RF_i_-rebound (‘rebound’) ribosomes. In the ‘first-bound’ case there is an initial activation step from ribosome state ${\rm{R}}_{\rm{P}}^{\rm{I}}$ with RF3-independent rate constant *k*_*i*IA_ that yields a rotation-activated ribosome complex ${\rm{R}}_{\rm{P}}^{\rm{A}}$ that can then undergo intersubunit rotation with rate constant ${\rm{k}}_{{\rm{iAR}}}^{\rm{ - }}$ in the absence and ${\rm{k}}_{{\rm{iAR}}}^{\rm{ + }}$in the presence of ribosome-bound RF3:


(4) $$\begin{equation*}\begin{array}{@{}l@{}} {\rm{R}}_{\rm{P}}^{\rm{I}} \cdot {\rm{R}}{{\rm{F}}}_{\rm{i}}{\rm{ + RF3}}\xrightarrow{{{{\rm{k}}}_{{\rm{iIA}}}}}{\rm{R}}_{\rm{P}}^{\rm{A}} \cdot {\rm{R}}{{\rm{F}}}_{\rm{i}}{\rm{ + RF3}}\xrightarrow{{{\rm{k}}_{{\rm{iAR}}}^{\rm{ - }}}}{\rm{R}}_{\rm{P}}^{\rm{R}}{\rm{ + R}}{{\rm{F}}}_{\rm{i}}{\rm{ + RF3}}\\ {\rm{ }} \downarrow \uparrow {{\rm{K}}}_3{\rm{ }}\hphantom{{\rm{R}}_{\rm{P}}^{\rm{I}} \cdot {\rm{R}}{{\rm{F}}}_{\rm{i}}{\rm{ + R}}\xrightarrow{{{{\rm{k}}}}}} \downarrow \uparrow {{\rm{K}}}_3{\rm{ }}\\ {\rm{R}}_{\rm{P}}^{\rm{I}} \cdot {\rm{R}}{{\rm{F}}}_{\rm{i}} \cdot {\rm{RF3}}\xrightarrow{{{{\rm{k}}}_{{\rm{iIA}}}}}{\rm{R}}_{\rm{P}}^{\rm{A}} \cdot {\rm{R}}{{\rm{F}}}_{\rm{i}} \cdot {\rm{RF3 }}\xrightarrow{{{\rm{k}}_{{\rm{iAR}}}^{\rm{ + }}}}{\rm{ R}}_{\rm{P}}^{\rm{R}} \cdot {\rm{R}}{{\rm{F}}}_{\rm{i}} \cdot {\rm{RF3}}\\ \xrightarrow{{{\rm{k}}_{{\rm{iRD}}}^{\rm{ + }}}}{\rm{R}}_{\rm{P}}^{\rm{R}} \cdot {\rm{RF3 + R}}{{\rm{F}}}_{\rm{i}} \end{array}\end{equation*}$$

**Figure 2.**
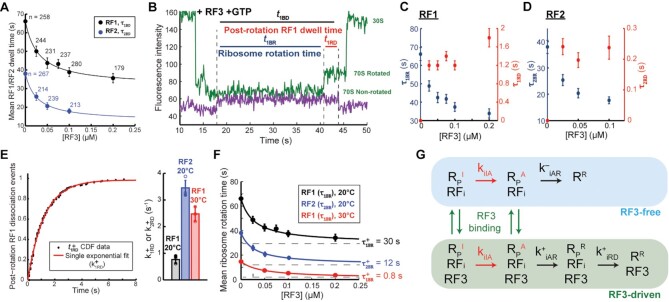
RF3 catalyzes intersubunit rotation to accelerate class-I RF dissociation in the presence of GTP. (**A**) Mean RF1 (${\tau }_{1BD}$) and RF2 (${\tau }_{2BD}$) dwell times as a function of [RF3] at 20°C in the presence of GTP, with reported sample size (*n*) for each dataset. Error bars are defined as S.E.M. (**B**) Representative trace of Cy5.5-RF1 terminating on Cy3B- and BHQ-2-labeled ribosome in the presence of dark RF3 and GTP. In the presence of GTP, RF3 induces a ribosome rotation before Cy5.5-RF1 dissociation, breaking the Cy5.5-RF1 dwell time into two time intervals defined as ribosome rotation time and post-rotation RF1 dwell time (${t}_{1BD} = {t}_{1BR} + {t}_{1RD}$). (**C**) Mean ribosome rotation time (${\tau }_{1BR}$) and mean post-rotation RF1 dwell time (${\tau }_{1RD}$) as a function of [RF3]. Sample sizes for these datapoints are reported in panel (A). Error bars are defined as S.E.M. (**D**) Mean ribosome rotation time (${\tau }_{2BR}$) and mean post-rotation RF2 dwell time (${\tau }_{2RD}$) as a function of [RF3]. Sample sizes for these datapoints are reported in panel (A). Error bars are defined as S.E.M. (**E**) Left: sample cumulative distribution of post-rotation RF1 dwell times ($t_{1RD}^ +$) fit to a single exponential function with rate constant, $k_{1RD}^ +$. Right: post-rotation RF1 ($k_{1RD}^ +$) and RF2 ($k_{2RD}^ +$) dissociation rate constants. Error bars are defined as SD with overlaid data points with sample size equal to the number of datasets collected under each condition in the presence of RF3. (**F**) Mean ribosome rotation times (${\tau }_{1BR}$ or ${\tau }_{2BR}$) as a function of [RF3] with data fit to Materials and Methods: Eq. (7) to determine the rate constant of [RF3]-independent activation. Sample sizes for the 20°C datapoints are reported in panel (A) and those for 30°C datapoints in increasing [RF3] order are *n* = 248, 237, 189 and 146. Error bars are defined as S.E.M. (**G**) Kinetic scheme describing an activation step with rate constant *k*_*i*IA_ that converts an inhibited RF1- or RF2-bound post-termination non-rotated ribosome R_P_·RF_i_ to an active state R_P_·RF_i_ before RF3 binds and accelerates ribosome intersubunit rotation to the R_P_^R^·RF_i_·RF3 state for subsequent RF1/RF2 dissociation with rate constant *k*^+^_1RD_ (also shown in Materials and Methods: Eq. (4)).

After intersubunit rotation, RF_i_ release occurs without detectable delay (<100 ms, as defined by the time-resolution of the instrument) in the absence and with detectable delay with mean time in the presence of RF3 (see Results). With experimental support for a sampling mode of RF3 binding (see Results) we assume initial equilibration with the same equilibrium dissociation constant *K*_3_ between RF3-free and RF3-containing complexes ${\rm{R}}_{\rm{P}}^{\rm{I}} \cdot {\rm{R}}{{\rm{F}}}_{\rm{i}}$ and ${\rm{R}}_{\rm{P}}^{\rm{A}} \cdot {\rm{R}}{{\rm{F}}}_{\rm{i}}$ (see the scheme Eq. 4). The [RF3] dependence of the mean duration time in Eq. (4) for subunit rotation,${\tau }_{iBR}$, counted from RF_*i*_-binding to appearance of rotated ribosome has then the form:


$$\begin{equation*}{\tau }_{iBR} = \frac{{\tau _{iBR}^ - {{\rm{K}}}_{\rm{3}} + \tau _{iBR}^ + \left[ {{\rm{RF3}}} \right]}}{{{{\rm{K}}}_{\rm{3}} + \left[ {{\rm{RF3}}} \right]}},\end{equation*}$$

where


(5) $$\begin{equation*}\tau _{iBR}^ - = \tau _{iIA}^ - + \tau _{iAR}^ - = \frac{1}{{{{\rm{k}}}_{{\rm{iIA}}}}} + \frac{1}{{{\rm{k}}_{{\rm{iAR}}}^{\rm{ - }}}},\end{equation*}$$

(6) $$\begin{equation*}\tau _{iBR}^ + = \tau _{iIA}^ + + \tau _{iAR}^ + = \frac{1}{{{{\rm{k}}}_{{\rm{iIA}}}}} + \frac{1}{{{\rm{k}}_{{\rm{iAR}}}^{\rm{ + }}}},{\rm{ }}\end{equation*}$$

so that


(7) $$\begin{equation*}{\tau }_{iBR} = \frac{{\rm{1}}}{{{{\rm{k}}}_{{\rm{iIA}}}}}{\rm{ + }}\frac{{{{\rm{K}}}_{\rm{3}}{\rm{ + }}\left[ {{\rm{RF3}}} \right]}}{{{\rm{k}}_{{\rm{iAR}}}^{\rm{ - }}{{\rm{K}}}_{\rm{3}}{\rm{ + k}}_{{\rm{iAR}}}^{\rm{ + }}\left[ {{\rm{RF3}}} \right]}}\end{equation*}$$

**Figure 3.**
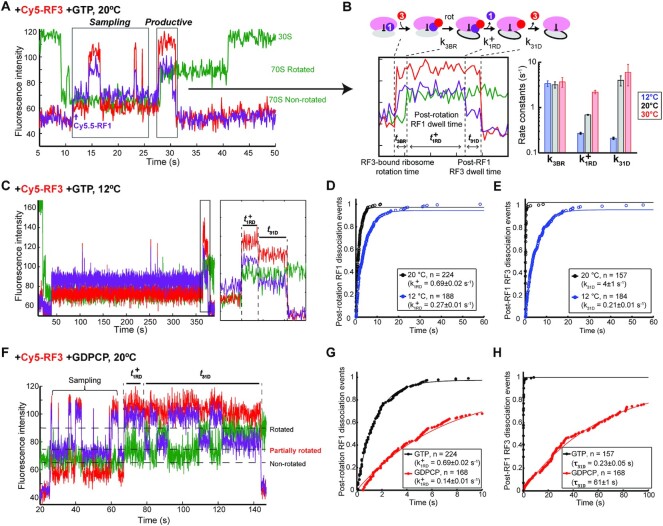
Productive RF3 binding results in ribosomal intersubunit rotation and sequential RF recycling events. (**A**) Representative trace of ribosome terminating on MF-UAA mRNA with Cy5.5-RF1, Cy5-RF3 and GTP at 20°C. After Cy5.5-RF1 binding, Cy5-RF3 binds to the ribosome either in sampling mode or productive mode, the latter coupled to ribosome rotation and subsequent RF dissociation events. (**B**) Left: representative trace of the experiment from panel (A) zoomed in to highlight the productive Cy5-RF3 event characterized by the sequential events of Cy5-RF3 binding, ribosome rotation, Cy5.5-RF1 dissociation, and Cy5-RF3 dissociation. The time intervals between these events are defined by stochastic parameters *t*_3BR_, *t*^+^_1RD_ and *t*_31D_. Right: RF3-bound ribosome rotation (*k*_3BR_), post-rotation RF1 dissociation ($k_{1RD}^ +$) and post-RF1 RF3 dissociation (*k*_31D_) rate constants, determined from single exponential fits to distributions of dwell times preceding the respective steps (*t*_3BR_, *t*^+^_1RD_ and *t*_31D_) at different temperatures. Error bars are defined as 95% CI. (**C**) Representative trace of ribosome terminating on MF-UAA mRNA with Cy5.5-RF1, Cy5-RF3 and GTP at 12°C, with the productive Cy5-RF3 binding boxed and zoomed in to the right. The zoomed-in trace outlines the timing of intersubunit rotation, Cy5.5-RF_1_ dissociation, and Cy5-RF3 dissociation used to measure the post-rotation RF1 dwell time ($t_{1RD}^ +$) and post-RF1 RF3 dwell time (${t}_{31D}$). (**D**) Cumulative distributions of post-rotation RF1 dwell times $t_{1RD}^ +$ at 20°C and 12°C. The distributions were fit to single exponential functions with rate constant $k_{1RD}^ +$. Errors are defined as 95% CI. (**E**) Cumulative distributions of post-RF1 RF3 dwell times ${t}_{31D}$ at 20°C and 12°C. The distributions were fit to single exponential functions with rate constant ${k}_{31D}$. Errors are defined as 95% CI. (**F**) Representative trace of ribosome terminating on MF-UAA mRNA with Cy5.5-RF1, Cy5-RF3 and GDPCP at 20°C (in the absence of RRF and EF-G). Cy5-RF3·GDPCP samples before its binding induces partial ribosome rotation. Post-rotation RF1 dwell time ($t_{1RD}^ +$) and post-RF1 RF3 dwell time (${t}_{31D}$) were measured as shown. (**G**) Cumulative distributions of post-rotation RF1 dwell times $t_{1RD}^ +$ in the presence of GTP and GDPCP. The distributions were fit to single exponential functions with rate constant $k_{1RD}^ +$. Errors are defined as 95% CI. (**H**) Cumulative distributions of post-RF1 RF3 dwell times ${t}_{31D}$ in the presence of GTP and GDPCP. The distributions were fit to single exponential functions with time constant ${\tau }_{31D}$. Errors are defined as 95% CI.

Analogously, according to the scheme in Eq. (4) the [RF3] dependence of the mean duration time ${\tau }_{iBD}$ from RF_*i*_-binding to RF_*i*_-dissociation is given by


(8) $$\begin{equation*}{\tau }_{iBD} = {\tau }_{iBR} + {\tau }_{iRD},\end{equation*}$$

where the post-rotation mean-dissociation time${\tau }_{iRD}$ is given by


(9) $$\begin{eqnarray*} {\tau }_{iRD} &=& \tau _{iRD}^ - \frac{{{{\rm{K}}}_{\rm{3}}}}{{{{\rm{K}}}_{\rm{3}}{\rm{ + }}\left[ {{\rm{RF3}}} \right]}} + \tau _{iRD}^ + \frac{{\left[ {{\rm{RF3}}} \right]}}{{{{\rm{K}}}_{\rm{3}} + \left[ {{\rm{RF3}}} \right]}}\nonumber\\ && = \tau _{iRD}^ + \frac{{\left[ {{\rm{RF3}}} \right]}}{{{K}_3 + \left[ {{\rm{RF3}}} \right]}} = \frac{1}{{k_{iRD}^ + }}\frac{{\left[ {{\rm{RF3}}} \right]}}{{{K}_3 + \left[ {{\rm{RF3}}} \right]}}\end{eqnarray*}$$

The latter two expressions follow from the experimental observation that $\tau _{iRD}^ - = 0$ (see Results).

In the ‘rebound’ case the ribosome is already activated at time zero and the scheme in Eq. (4) is replaced by


(10) $$\begin{equation*}\begin{array}{@{}l@{}} {\rm{R}}_{\rm{P}}^{\rm{A}} \cdot {\rm{R}}{{\rm{F}}}_{\rm{i}}{\rm{ + RF3}}\xrightarrow{{{\rm{k}}_{{\rm{iAR}}}^{\rm{ - }}}}{\rm{R}}_{\rm{P}}^{\rm{R}}{\rm{ + R}}{{\rm{F}}}_{\rm{i}}{\rm{ + RF3}}\\ {\rm{ }} \downarrow \uparrow {{\rm{K}}}_3{\rm{ }}\\ {\rm{R}}_{\rm{P}}^{\rm{A}} \cdot {\rm{R}}{{\rm{F}}}_{\rm{i}} \cdot {\rm{RF3 }}\xrightarrow{{{\rm{k}}_{{\rm{iAR}}}^{\rm{ + }}}}{\rm{R}}_{\rm{P}}^{\rm{R}} \cdot {\rm{R}}{{\rm{F}}}_{\rm{i}} \cdot {\rm{RF3}}\xrightarrow{{{\rm{k}}_{{\rm{iRD}}}^{\rm{ + }}}}{\rm{R}}_{\rm{P}}^{\rm{R}} \cdot {\rm{RF3 + R}}{{\rm{F}}}_{\rm{i}}\\ {\rm{ }} \end{array}\end{equation*}$$

In Eqs. (5) and (7), we set ${\tau }_{iIA}$equal to zero while the forms of Eqs. (8) and (9) are unchanged.

The algebraic expression in Eq. (7) was fit to the mean ribosome rotation data shown in Figure 2F and Figure S2D, with the ${\rm{k}}_{{\rm{iAR}}}^{\rm{ + }}$fixed to 3 s^−1^ based on the rate constant of RF3-bound ribosome rotation (k_3BR_) measured from the single-molecule data collected in the presence of Cy5-RF3 (see Results).

### Intracellular kinetic flow modeling

#### Relation between bacterial population doubling time T*_d_* and average translation cycle time ***τ_R_***

The rate dM/dt of increase of the number M of peptide bonds per time unit in the cell population is given by:


(11) $$\begin{equation*}\frac{{dM}}{{dt}} = {v}_RN_R^{UAA}\frac{{{\tau }_E}}{{\tau _R^{UAA}}} + {v}_RN_R^{UGA}\frac{{{\tau }_E}}{{\tau _R^{UGA}}} + {v}_RN_R^{UAG}\frac{{{\tau }_E}}{{\tau _R^{UAG}}}\end{equation*}$$

Here, $N_R^{UAA}$, $N_R^{UGA}$ and $N_R^{UAG}$ are the numbers of ribosomes in the cell population translating mRNA with UAA, UGA and UAG stop codons, respectively; ${v}_R$is the elongation speed of the ribosome in number of peptide bonds per elongating ribosome per second, ${\tau }_E$ is the time the ribosome spends in the elongation mode of the translation cycle elongating peptide of 300 AAs average length. Total translation cycle times for ribosomes translating mRNAs with UAA, UGA and UAG stop codons are $\tau _R^{UAA}$, $\tau _R^{UGA}$ and $\tau _R^{UAG}$, respectively; they include times for initiation, elongation (${\tau }_E$), termination and recycling. Accordingly, each ratio ${\tau }_E/\tau _R^X$ in Eq. (11) is the fraction of ribosomes in elongation mode translating mRNAs with stop codon X. Introducing fractions $f_R^X$ of ribosomes translating mRNAs with stop codons X and normalizing by the current number of peptide bonds, *M*, Eq. (11) can be re-written as:


(12) $$\begin{equation*}\frac{1}{M}\frac{{dM}}{{dt}} = {v}_R\frac{{{N}_R}}{M}\frac{{{\tau }_E}}{{{\tau }_R}}\end{equation*}$$

Here, ${N}_R$ is the total number of ribosomes in the cell population; ***τ_R_*** is the average translation cycle time per ribosome given by:


(13) $$\begin{equation*}{\tau }_R = 1/\left\{ {\frac{{f_R^{UAA}}}{{\tau _R^{UAA}}} + \frac{{f_R^{UGA}}}{{\tau _R^{UGA}}} + \frac{{f_R^{UAG}}}{{\tau _R^{UAG}}}} \right\}\end{equation*}$$

In the steady state, the ratio $r = {N}_R/M$ between total number of ribosomes (*N_R_*) and peptide bonds (*M*) in the cell population is constant. The increase in M (which is proportional to cell population biomass) is given by:


(14) $$\begin{equation*}M = {M}_0\exp (A\frac{t}{{{\tau }_R}}),\end{equation*}$$

where constant $A = {v}_R{\tau }_E{N}_R/M = {v}_R{\tau }_Er$. From Eq. 14 it is seen that bacterial population doubling time *T_d_* is proportional to ***τ_R_*** and given by:


(15) $$\begin{equation*}{T}_d = {\tau }_R\frac{{\ln (2)}}{A}.\end{equation*}$$

### Bulk kinetics assays

#### Components and buffers

Buffers and *E. coli* components for cell-free protein synthesis were prepared as described (7). Reaction buffer was polymixṇHEPES pH 7.5, containing 5 mM Mg(OAc)_2_, 95 mM KCl, 3 mM NH_4_Cl, 0.5 mM CaCl_2_, 1 mM spermidine, 8 mM putrescine, 1 mM dithioerythritol and 30 mM Hepes. It was supplemented with an energy regeneration system containing 2 mM GTP, 10 mM phosphoenolpyruvate, 50 μg/ml pyruvate kinase and 2 μg/ml myokinase. Purified ribosomal release complexes (RC) were prepared as described (8). RF1 had methylated Glu in the catalytic Gly-Gly-Glu (GGQ) motif and was purified as described (8). The total RF1 concentration was determined by Bradford assay and the concentration of active RF1 was determined by the amount of peptide released during a single round of termination with RC in excess over RF1 (12). Both RC and RF1 samples were pre-incubated for 1 min at 37°C prior to the reaction. 112 pmol RC were mixed with 0 - 100 pmol RF1 for 15 s at 37°C (reaction volume 70 μl) and quenched with 17% (final concentration) formic acid. RF3 and energy regeneration system were not included to avoid recycling of RF1. The quenched samples were treated as described for quench flow experiments (8). RF3 was overexpressed from an osmo-expression vector and further purified with ion exchange and gel filtration chromatography (14).

#### Peptide release

Peptide release experiments were performed by stopped flow technique at 20°C, essentially as described (8). RC contained Met-Phe-Phe-tRNA^Phe^ in the P site and UAA stop codon in the A site. The peptide was labeled with a fluorescent coumarin derivative (7-methoxycoumarin-3-carboxylic acid *N*-succinimidyl ester) and tritium (^3^H) on methionine. 0.05 μM RC were reacted to a saturating concentration of RF1 (3.4 μM) or 3.4 μM RF1 and 8 μM RF3.

#### Recycling of RF1 by RF3

RF1 recycling assays were performed at 20°C with RC in large excess over RF1, so that complete termination would require multiple rounds of RF1 recycling by RF3. RC contained fMet-Phe-Phe-tRNA^Phe^ labeled with ^3^H on methionine in the P site and UAA stop codon in the A site. RC and RF1 reaction mixes were pre-incubated for 5 min at 20°C, then 5 pmol RC were reacted to 0.1 pmol RF1 or 0.1 pmol RF1 and 25 pmol RF3. Aliquots were taken by hand at different time points and quenched with 17% (final concentration) formic acid. The amount of released peptide was determined by scintillation counting as described (8). The time per cycle $\tau$ was calculated by dividing the amount of RF1 per aliquot (pmol) by the slope of a regression line (pmol/s) of the peptide released over time during the initial linear phase of the reaction (17).

## RESULTS

### RF3 accelerates ribosome intersubunit rotation rate for rapid class-I RF dissociation

To explore the dynamics of RF3-ribosome interaction in the presence of GTP, we measured the timing of ribosome intersubunit rotation during termination of protein synthesis in the context of the entire mRNA translation cycle.

We applied single-molecule fluorescence assays using zero-mode waveguide (ZMW)-based instrumentation (39). The intersubunit rotational movements during initiation, elongation, termination and recycling stages of the ribosomal protein synthesis were monitored by site-specifically labeled 30S and 50S subunits with Cy3B and BHQ-2 (a non-fluorescent quencher), respectively, allowing for FRET between the two dyes (40). The arrival and departure of fluorescently-labeled release factors during translation termination were monitored in real time (18).

Translation was started by delivering BHQ-50S subunit, elongation/recycling mix containing aa-tRNA, EF-Tu, EF-G, RRF, GTP and Cy5.5-labeled RF1 or RF2 to immobilized Cy3B-30S preinitiation complexes (30S subunit–mRNA–initiator tRNA) (Figure 1A). Translation initiation was signaled by quenching of Cy3B-30S (initially high Cy3B intensity) by BHQ-50S upon subunit joining to form the 70S ribosome in non-rotated state (low Cy3B intensity). Translation elongation cycles were signaled by Cy3B fluorescence changes due to transitions between the non-rotated (low Cy3B intensity) and rotated (medium Cy3B intensity) states of the 70S ribosome. Eventually, the arrival of a stop codon into the A site was signaled by the Cy5.5 fluorescence increase due to A-site binding of a Cy5.5-labeled class-I RF. Departure of Cy5.5-labeled class-I RF was observed as Cy5.5 fluorescence decrease which, in the absence of RF3, coincides with Cy3B fluorescence increase due to ribosome intersubunit rotation (Figure 1B). After the rotation and release factor dissociation, the 70S ribosome was split into subunits by RRF and EF-G (41), resulting in de-quenching of Cy3B signal.

Mean dwell time ${\tau }_{iBD}$ for RF1 or RF2 (RF_i_, *i* = 1 or 2) on the terminating ribosome, defined as the mean of the time between RF_i_ binding to and dissociation from the ribosome, was estimated as $\tau _{1BD}^ -$ = 66 ± 3 s for RF1 and $\tau _{2BD}^ -$ = 38 ± 2 s for RF2 in the absence of RF3 (specified with a minus) (see Materials and Methods for details). These values coincide with the mean ribosome rotation time ${\tau }_{iBR}$ between RF_i_ binding and ribosomal intersubunit rotation ( $\tau _{iBD}^ -$=$\tau _{iBR}^ -$ ) since all RF1 and RF2 dissociation events occur concurrently with ribosome rotation in the absence of RF3 (Figure 1C). Increasing the unlabeled RF3 concentration decreased ${\tau }_{iBD}$for RF1 and RF2 (Figure 2A), consistent with a previously observed RF3-dependent increase of RF_i_ dissociation rate from A site in GTP presence (14). Addition of RF3 introduced a short post-rotation stochastic delay time conditional on that the ribosome is RF3-bound between ribosome rotation and RF_i_ release, $t_{iRD}^ +$, (Figure 2B) with mean value $\tau _{iRD}^ +$. For both RF1 and RF2, with increasing [RF3] the mean ribosome rotation time ${\tau }_{iBR}$ decreased (Materials and Methods: Eq. 7) and mean post-rotation RF_i_ dwell time $\tau _{iRD}^{}$, defined as the probability that the ribosome is free from RF3 multiplied with $\tau _{iBD}^ - ( = 0)$ plus the probability that the ribosome is RF3 bound multiplied with $\tau _{iRD}^ +$ (Materials and Methods: Eq. 9) increased from zero toward its plateau value $\tau _{iRD}^ +$ (Figure 2C, D). From these observations we conclude that (i) in the GTP presence RF3 accelerated RF_i_ dissociation by inducing ribosome rotation, and (ii) intersubunit rotation subsequently induced rapid RF_i_ dissociation from the ribosome. From the subset of ribosomes that display the post-rotation stochastic delay $t_{iRD}^ +$ of RF_i_ dissociation, we fitted the distribution of $t_{iRD}^ +$ to a single exponential function (Materials and Methods: Eq. 1) and estimated rate constants for RF1 and RF2 dissociation from the rotated ribosome as ${\rm{1/}}\tau _{1RD}^ + {\rm{ = k}}_{1RD}^ +$= 0.76 ± 0.04 s^−1^ and ${\rm{1/}}\tau _{2RD}^ + {\rm{ = k}}_{2RD}^ +$= 3.5 ± 0.4 s^−1^ at 20°C, respectively (Figure 2E, Table 1). Fitting Materials and Methods: Eq. (7) to the [RF3] dependence of ${\tau }_{iBR}$ (Figure 2F) revealed surprisingly long rotation times $\tau _{iBR}^ +$ at saturating [RF3]: at 20°C the $\tau _{iBR}^ +$ estimates for RF1 and RF2 are 30 s and 12 s, respectively, and at 30°C the $\tau _{1BR}^ +$ value is 0.8 s . The $\tau _{iBR}^ +$ value for RF1 at 20°C agrees with the RF1 recycling time of 23.5 ± 2 s (k_cat_ = 0.04 s^−1^) at 20°C measured from a bulk RF1 recycling assay at saturating [RF3] (Supplementary Figure S1A).

**Table 1.** Kinetic parameters of RF1/RF2 termination on MF-UAA measured with WT RF3

Description	Rate constant	RF1 at 20°C	RF1 at 30°C	RF2 at 20°C
RF1/RF2 association rate to pre-termination 70S complex	*k*_ion_	21 ± 2 μM^−1^s^−1^	-	10.5 μM^−1^s^−1 ‡^
[RF3]-independent activation rate of post-termination ribosome	*k*_iIA_	0.034 ± 0.007 s^−1^	1.3 ± 0.3 s^−1^	(0.09 s^−1^)*
Spontaneous and simultaneous RF1/RF2 dissociation and ribosome rotation rate from post-termination ribosome	$k_{iAR}^ -$	0.027 ± 0.005 s^−1^	0.072 ± 0.001 s^−1^	0.04 ± 0.03 s^−1^
RF1/RF2 dissociation rate from RF3-induced rotated post-termination ribosome	$k_{iRD}^ +$	0.76 ± 0.14 s^−1^	2.5 ± 0.3 s^−1^	3.5 ± 0.4 s^−1^

*Numbers in parentheses have 95% CI that spans negative values.

^‡^RF2 association kinetics on UAA codon referenced from Prabhakar *et al.*, 2017.

To investigate further the slow intersubunit rotation at saturating [RF3], we took advantage of the observation that in the absence of RRF a post-termination ribosome from which a ‘first-bound’ RF_i_ has dissociated can readily rebind another RF_i_ (15). This brings the ‘rebound’ ribosome back to the non-rotated intersubunit state (18), as also observed here with Cy5.5-RF1 (Supplementary Figure S2A). We found that the $\tau _{1BR}^{}$times and their responses to RF3 addition are remarkably different for ‘first-bound’ and ‘rebound’ ribosomes (Supplementary Figure S2D). Fits of these data to Materials and Methods: Eq. (7) lend strong support to the hypothesis that ‘first-bound’ ribosome rotation is initially blocked and therefore occurs in two consecutive steps: an activation step which is insensitive to RF3 presence (${{\rm{k}}}_{{\rm{iIA}}}$), a slow (${\rm{k}}_{{\rm{iAR}}}^{\rm{ - }}$) and a fast (${\rm{k}}_{{\rm{iAR}}}^{\rm{ + }}$) rotation step in absence and presence of RF3, respectively (Figure 2G; Materials and Methods: Eq. 4). In contrast, the ‘rebound’ ribosomes appear already activated, so that rotation occurs in a single step (Materials and Methods: Eq. 9). Accordingly, for ‘first-bound’ ribosomes we estimate ${\rm{k}}_{{\rm{iIA}}}^{}$and ${\rm{k}}_{{\rm{iAR}}}^{\rm{ - }}$ as 0.03 s^−1^ and 0.04 s^−1^, respectively. For ‘rebound’ ribosomes we estimate ${\rm{k}}_{{\rm{iAR}}}^{\rm{ - }}$= 0.08 s^−1^, a value similar to the first time bound estimate of 0.04 s^−1^, in line with the model (Figure 2G, Materials and Methods: compare Eq. 4 with Eq. 10).

Further, RF1 dissociation times from ‘first time bound’ and ‘rebound’ ribosomes coincide with stochastic ribosome rotation times in absence $(t_{1BD}^ - = t_{1BR}^ - ){\rm{ }}$ but not in the presence of RF3, where dissociation is delayed in relation to rotation ($t_{iBD}^ + = t_{iBR}^ + + t_{iRD}^ +$) (Supplementary Figure S2B). Here, in the presence of RF3, these dwell times $t_{iRD}^ +$ of the ‘first-bound’ and ‘rebound’ RF1 are exponentially distributed with rate constants ${\rm{k}}_{{\rm{1RD}}}^{\rm{ + }}$ estimated as 0.77 ± 0.11 s^−1^ and 1.0 ± 0.2 s^−1^, respectively, similar to ${\rm{k}}_{{\rm{1RD}}}^{\rm{ + }}$= 0.87 ± 0.04 s^−1^ for the single RF1 binding event in the presence of RRF (Supplementary Figure S2C).

Applying the same model (Figure 2G; Materials and Methods: Eqs. 4, 7) to ‘first-bound’ kinetics in the presence of RRF, we estimate the ribosome activation rate constant to be ${{\rm{k}}}_{{\rm{1IA}}}$ = 0.034 s^−1^ at 20°C and 1.3 s^−1^ at 30°C (Table 1), representing the plateau values in Figure 2F. The 38-fold increase in ${{\rm{k}}}_{{\rm{1IA}}}$ at 30°C indicates a high enthalpic contribution to the standard free energy barrier for activation of intersubunit rotation. Rate constant ${{\rm{k}}}_{{\rm{1IA}}}$ remains small (0.07 s^−1^) at 20°C even for termination on longer peptides (13 aa), suggesting physiological relevance of this reaction barrier as a growth inhibitor at low but not high temperatures (Supplementary Figure S2E-F). Natural guesses of its origin would be hydrolysis of the peptidyl-tRNA ester bond or the subsequent peptide dissociation, suggested to precede subunit rotation and class-I RF dissociation (12). However, our bulk kinetic data show that the ester bond hydrolysis induced by RF_i_ and subsequent dissociation of fluorescently-labeled peptide from the ribosome occur too fast (2 s^−1^) to match ${{\rm{k}}}_{{\rm{1IA}}}$ at 20°C (Supplementary Figure S1B), thus the origin of this rotation blockage remains unknown (Discussion).

### RF3-catalyzed intersubunit rotation exhibits complex dynamics

To reveal the temporal relation of the slow ribosome rotation at 20°C (Figure 2F, G) to the binding and dissociation of RF3, we next directly monitored RF3 occupancy simultaneously with RF1 binding and ribosome rotation during termination using Cy5-labeled RF3. In the presence of GTP, two distinct modes of Cy5-RF3 binding were observed on ribosomes bound to Cy5.5-RF1 during termination (Figure 3A). The first mode, denoted the ‘sampling mode’, shows RF3 binding and dissociation events with no change in RF1 occupancy or ribosome conformation. The second mode, denoted as ‘productive mode’ that followed the ‘sampling mode’ in most recorded traces (like in Figure 3A), shows RF3 binding followed by a rapid ribosome rotation (*k*_3BR_ = 3.2 ± 0.5 s^−1^) for 51 ± 2% of the ribosomes while the remaining ribosomes apparently rotated concurrently with productive RF3 binding. The apparent ‘concurrency’ of the two events is due, most probably, to the two events occurring with a delay time less than the time resolution of the measurement (100 ms). Ribosomal intersubunit rotation was followed by Cy5.5-RF1 dissociation (${\rm{k}}_{{\rm{1RD}}}^{\rm{ + }}$ = 0.69 ± 0.02 s^−1^) and then by Cy5-RF3 dissociation (*k*_31D_ = 4 ± 1 s^−1^) for 68 ± 3% of the ribosomes (Figure 3B). As much as 31 ± 1% of the ribosomes show apparently simultaneous dissociation of RF1 and RF3, a result, we suggest, due to the limited time resolution of the measurement. The remaining small (<5%) fraction shows early disappearance of Cy5-RF3 signal before Cy5.5-RF1 dissociation. These results support a sequential RF dissociation model where ribosome rotation triggers RF1 dissociation followed by rapid RF3 dissociation. In line with this model, lowering the temperature further (to 12°C) extends the fraction of ribosomes following this sequential dissociation pathway to 98%, accompanied by a 2.6-fold slow-down in RF1 dissociation and a 21-fold slow-down in RF3 dissociation (Figure 3C-E).

Termination in the presence of the non-hydrolysable GTP analogue GDPCP shows that Cy5-RF3 can still bind productively to the RF1-ribosome complex and catalyze intersubunit rotation, but Cy5-RF3∙GDPCP is afterward trapped on the rotated ribosome (Figure 3F). The intersubunit rotation following Cy5-RF3 binding in GDPCP presence occurs through an interim ribosome conformational state represented by an intermediate Cy3B-BHQ intensity (Supplementary Figure S3). This interim state likely represents a partial rotation of the ribosome that was previously revealed in a cryo-EM structure of a partially rotated ribosome bound to RF1 and RF3∙GDPCP (29). The Cy5.5-RF1 then dissociates from this intermediate state 4.6-fold more slowly than from the RF3∙GTP-induced rotated state (Figure 3G). This Cy5.5-RF1 dissociation event is coupled to full ribosome rotation (Supplementary Figure S3). The rapid dissociation of Cy5-RF3 after RF1 departure in the presence of GTP is contrasted by the 270-fold slower dissociation of Cy5-RF3 in the presence of GDPCP (Figure 3H). These observations suggest that the primary role of GTP hydrolysis on RF3 is to promote its rapid dissociation after intersubunit rotation and RF1 dissociation.

In presence of GTP, the addition of the antimicrobial peptide Api137 eliminated RF3-accelerated intersubunit rotation and extended the Cy5.5-RF1 occupancy time to the Cy5.5 photobleaching and movie-time limit (300 ± 7 s) (Supplementary Figure S4A-B), while the rates of elongation and Cy5.5-RF1 arrival were unaffected (Supplementary Figure S4C–F). This resulted in perpetual cycles of Cy5-RF3 sampling without intersubunit rotation throughout the entire movie (6 min) (Supplementary Figure S4G, H), suggesting that Api137 inhibits RF3-mediated intersubunit rotation by thermodynamically and/or kinetically stabilizing the RF1-bound non-rotated ribosomal state.

**Figure 4.**
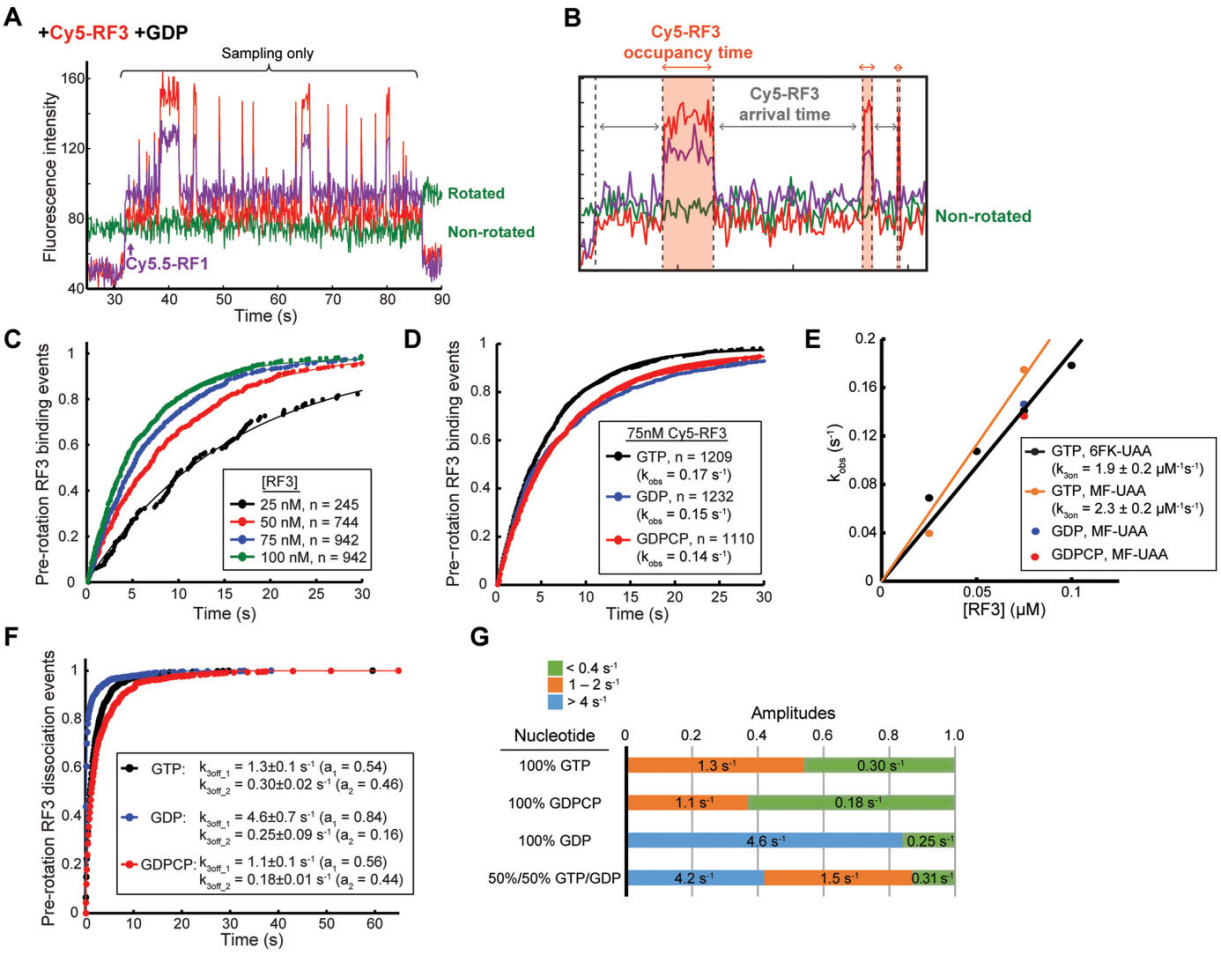
RF3 sampling dynamics is modulated by guanine nucleotide identity. (**A**) Representative trace of ribosome terminating on MF-UAA mRNA with Cy5.5-RF1, Cy5-RF3 and GDP at 20°C. In the presence of GDP, Cy5-RF3 only samples the Cy5.5-RF1-bound ribosome with no subsequent ribosome intersubunit rotation during Cy5-RF3 occupancy, indicating the absence of a productive binding mode. (**B**) Representative trace from the experiment in Figure 3A zoomed in to highlight the Cy5-RF3 sampling events in the presence of GTP. A Cy5-RF3 occupancy time is defined here as the time interval between a Cy5-RF3 binding and subsequent dissociation event, and a Cy5-RF3 arrival time as the time interval between the Cy5.5-RF1 binding or a Cy5-RF3 dissociation event and subsequent Cy5-RF3 binding event. (**C**) Cumulative distributions of Cy5-RF3 arrival times at different RF3 concentrations in the presence of GTP for 6FK-UAA mRNA. These distributions were fit to single exponential functions. (**D**) Cumulative distributions of Cy5-RF3 arrival times at 75nM Cy5-RF3 concentration in the presence of GTP, GDP, or GDPCP for MF-UAA mRNA. These distributions were fit to single exponential functions. (**E**) Plot of observed rate constants *k*_obs_ determined from single exponential fits in panels (C) and (D) as a function of [RF3] with linear fits to determine bimolecular association rate constants. (**F**) Cumulative distributions of Cy5-RF3 occupancy times before ribosome rotation in the presence of GTP, GDP, and GDPCP. These distributions were fit to double exponential functions. Errors are defined as 95% CI. (**G**) Distribution of amplitudes determined from double or triple exponential fits to cumulative distributions of Cy5-RF3 occupancy times under different guanine nucleotide conditions. The numbers reported are the RF3 dissociation rate constants (*k*_3off1_, *k*_3off2_, *k*_3off3_) extracted from the fits.

We next determined the effects of different nucleotides GTP, GDP, GDPCP and their mixtures on RF3 binding dynamics. The productive Cy5-RF3 binding events that are coupled to intersubunit rotation and RF1 dissociation—as observed in the presence of GTP or GDPCP (Figure 3A, F)—were not observed in the presence of GDP (Figure 4A), confirming that RF3-induced intersubunit rotation strictly requires GTP or one of its analogues. In contrast, the sampling Cy5-RF3 events prior to the productive event were observed in the presence of GTP, GDPCP, or GDP (Figure 3A, F; Figure 4A), showing this initial sampling phenomenon to be GTPase-independent. The time intervals measured between the Cy5.5-RF1 binding and Cy5-RF3 binding or between the Cy5-RF3 dissociation and subsequent Cy5-RF3 binding events in the sampling mode, termed here arrival times (see Figure 4B), are exponentially distributed (Figure 4C,D). From the exponential factors *k*_obs_ of these time distributions a bimolecular association rate constant (*k*_3on_) can be obtained as *k*_obs_/[RF3] at any RF3 concentration. In the presence of GTP, *k*_3on_ was estimated as 2.3 ± 0.2 μM^−1^s^−1^ (Figure 4E, Table 2), and very similar *k*_3on_ values were estimated from the arrival time distributions measured at 75 nM RF3·GDP or RF3·GDPCP concentration (Figure 4D, E). This suggests similar and rapid association kinetics for the GDP, GDPCP and GTP forms of RF3, fully compatible with the previously observed, rapid GDP to GTP exchange on RF3 in complex with the class-I RF bound ribosome (16,23).

**Table 2.** Kinetic parameters of RF1 termination on MF-UAA measured with Cy5-RF3

Description	Rate constant	GTP	GDP	GDPCP
RF3 association rate to post-termination RF1-70S complex	*k*_3on_	2.3 ± 0.2 μM^−1^s^−1^	2 μM^−1^s^−1^	2 μM^−1^s^−1^
Sampling RF3 dissociation rates from non-rotated	*k*_3off1_	1.3 ± 0.1 s^−1^	4.6 ± 0.7 s^−1^	1.1 ± 0.1 s^−1^
RF1-RF3-70S complex		(*a*1 = 0.54)	(*a*1 = 0.84)	(*a*1 = 0.56)
	*k*_3off2_	0.30 ± 0.02 s^−1^	0.25 ± 0.09 s^−1^	0.18 ± 0.01 s^−1^
		(*a*2 = 0.46)	(*a*2 = 0.16)	(*a*2 = 0.44)
RF3-bound ribosome rotation rate.	*k*_3BR_	3.2 ± 0.5 s^−1^	-	2.1 ± 0.2 s^−1^
RF1 dissociation rate from rotated RF1-RF3-70S complex.	$k_{1RD}^ +$	0.69 ± 0.02 s^−1^	-	0.14 ± 0.01 s^−1^
RF3 dissociation rate from rotated RF3-70S complex.	*k*_31D_	4 ± 1 s^−1^	-	0.0164 ± 0.004 s^−1^

The occupancy times defined as time intervals between binding and subsequent departure of Cy5-RF3 in sampling mode (Figure 4B) reveal a biphasic distribution (Figure 4F), with a fast phase dissociation rate (*k*_3off,1_) of 1.3 ± 0.1 s^−1^ and slow phase dissociation rate (*k*_3off,2_) of 0.30 ± 0.02 s^−1^ in the presence of GTP (distribution was fit to a double exponential, Materials and Methods: Eq. 2) (Figure 4F, G). This suggests that in the presence of GTP, RF3 can bind to the non-rotated RF1-bound ribosome in two distinct states with different affinities.This biphasic behavior persists in the presence of GDPCP and GDP. With GDPCP the fast phase rate is very similar to that with GTP while with GDP the fast phase rate is 4.6±0.3 s^-1^, i.e. 3.5-fold that with GTP (Figure 4F, G). These distinct fast phases identified in the presence of 100% GTP and 100% GDP were both present when fitting a triple exponential (Materials and Methods: Eq. 3) to the Cy5-RF3 occupancy time distribution in the presence of 50% GDP (1:1 ratio of GTP:GDP) (Figure 4G), with the slowest phase having a similar rate (about 0.3 s^−1^) as the slow phases of the 100% GTP and 100% GDP distributions. While the fast >4 s^−1^ phase confirms the existence of a shorter-lived RF3·GDP state on the ribosome, the consistent occurrence of the slowest phase with a dissociation rate constant less than 0.4 s^−1^ suggests the existence also of a higher affinity RF3 state on the non-rotated ribosome that is not dependent on which guanine nucleotide is present. We propose that this high-affinity state represents the apo-RF3 intermediate induced by the ribosome to facilitate the GDP-to-GTP exchange mechanism (see SI for explanation of the observed states).

### Kinetic modeling of intracellular termination flows

**Figure 5.**
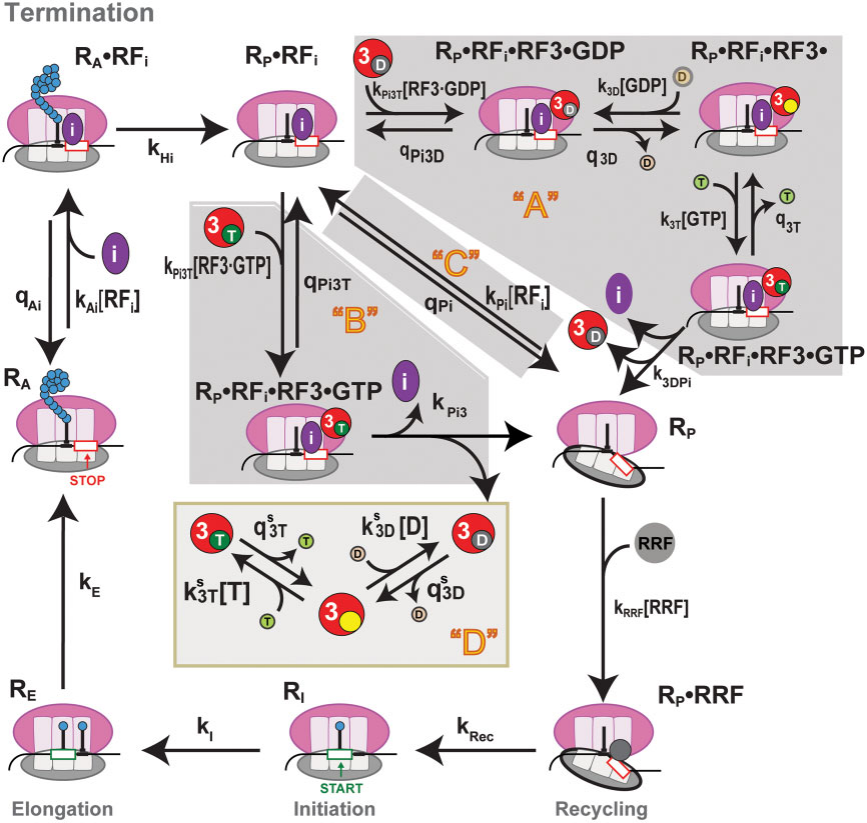
Scheme for ribosomal protein synthesis in the bacterial cell. In termination phase of protein synthesis class-I RFs (RF_i_) bind to the stop codon programmed A site of pre-termination ribosome (R_A_) leading to the formation of ribosomal complex R_A_·RF_i_ (upper left corner of Figure 5), upon which the RF_i_-induced peptide release from peptidyl-tRNA in P site leads to post-termination complex (R_P_·RF_i_). Subsequent RF_i_ and RF3 release via three alternative pathways (upper right part of Figure 5) eventually leads to post termination ribosome (R_P_). Termination ends by irreversible splitting of R_P_ into ribosomal subunits by ribosome recycling factor RRF and elongation factor G (EF-G) (41,51,52). Subsequently, ribosomal subunits are recycled to initiation complex (R_I_) (53,54) leading to elongation complex (R_E_) with the global cycle of protein synthesis closed by the formation of pre-termination ribosome R_A_ (see SI for details).

The rational for the kinetic modeling of intracellular termination flows in the present work (Figure 5) is to answer pertinent and yet unanswered questions regarding RF3 function. One such question is if, in the living cell, the biochemically well-established guanine nucleotide exchange factor activity of the ribosome (16,23,42) is required for RF3$ \cdot$GDP conversion to its active RF3$ \cdot$GTP form (12,23,42) or, as suggested by Peske *et al.* (16) the spontaneous conversion of RF3$ \cdot$GDP to RF3$ \cdot$GTP in the cytoplasm is sufficient to assure the rate of RF3$ \cdot$GTP supply that matches the rate of demand of activated RF3$ \cdot$GTP in termination of protein synthesis. Another pertinent question is if rapid dissociation of RF2 in the absence of RF3 (24) explains the small growth rate reduction by RF3 deficiency in some bacterial strains (43).

Accordingly, we used kinetic modeling to decide if the formation of active RF3·GTP occurs mainly through GDP to GTP exchange on free RF3 in bulk solution (16) or on ribosome-bound RF3 (23). We used *in vitro* estimates of kinetic parameters for the interactions of class-I RFs and RF3 to model translation termination embedded in the entire protein synthesis cycle (Figure 5). We focused on the kinetic coupling between the action cycles of RF1/RF2 and RF3 on one hand, and the action cycles of peptide release from the ribosome on the other hand (Figure 5, Supplementary Figure S6A).

There are three alternative pathways (‘A’, ‘B’ and ‘C’) for RF1 and RF2 (for brevity denoted as RF_i_ where *i* = 1 or 2) release from post-translation ribosomal complex R_P_·RF_i_ (Figure 5). There are also two pathways for GDP to GTP exchange on RF3: one spontaneous ‘off’ the ribosome in solution (Figure 5, pathway ‘D’) and one ‘on’ the ribosome (Figure 5, pathway ‘A’). In the latter pathway RF3·GDP binds to post-termination complex R_P_·RF_i_ to form an R_P_·RF_i_·RF3·GDP complex. Rapid GDP dissociation from this ribosome-bound RF3·GDP (16) results in an R_P_·RF_i_·RF3· intermediate complex containing nucleotide-free, apo-RF3 (RF3·) which, upon GTP binding, becomes the activated ribosome-bound RF3·GTP in the R_P_·RF_i_·RF3·GTP complex (Figure 5). This activated ribosome-bound RF3·GTP catalyzes RF_i_ dissociation, which is followed by rapid GTP hydrolysis on RF3·GTP leading to RF3·GDP formation and dissociation from the ribosome, resulting in post-termination complex R_P_ with unoccupied A site ready to bind RRF (12,23,26,28). In pathway B, RF3·GTP formed by spontaneous GDP to GTP exchange in solution binds to post-termination complex R_P_·RF_i_ directly leading to complex R_P_·RF_i_·RF3·GTP and from there the reactions are as in pathway A (16,24). Importantly, pathway B requires spontaneous GDP dissociation from RF3·GDP ‘off’ the ribosome (Figure 5, Rectangle ‘D’, arrow denoted q^S^_3D_), a slow reaction known to be rate limiting for the spontaneous GDP-to-GTP exchange on RF3 off the ribosome. In pathway C, release factor RF_i_ dissociates from post-termination complex R_P_·RF_i_ in an RF3-independent manner as in RF3-deficient *E. coli* mutants (19,43) and other RF3-lacking bacteria (44).

For actual modeling the detailed reaction scheme in Figure 5 is simplified to the scheme in Supplementary Figure S6B with identical steady state properties. In the case of termination on UAA codons read by both RF1 and RF2 the scheme in Supplementary Figure S7 was used. Steady-state values of flows and concentrations of ribosomal complex and free factors in Supplementary Figure S6B and Supplementary Figure S7 were derived from ordinary differential equations (see SI for details). All kinetic parameters used in the modeling are compiled in Supplementary Table S1 along with their sources.

Following known frequencies of stop codons (45), modeling was conducted with ribosomes translating mRNAs with stop codons UAA (64% read by RF1 or RF2), UGA (27%, read by RF2) and UAG (9%, read by RF1). At an elongation rate of 20 codons (amino acids) per second, ribosomes (20 μM total concentration) spend 15 s synthesizing an average size protein of 300 amino acid residues (46). In the absence of RF3, RF1 dissociates from the post-termination ribosome more slowly than RF2 (0.1 s^−1^ versus 1 s^−1^, respectively) (Supplementary Table S1). In our modeling the free GDP concentration was set to zero, a value maximizing the GDP-dependent part of the off ribosome rate of regeneration of RF3$ \cdot$GTP from RF3$ \cdot$GDP. In this limit, regeneration depends on the rate constant, q^S^_3D_, for spontaneous dissociation of GDP from RF3·GDP as well as on the free GTP concentration, set to 0.5 mM (see SI).

**Figure 6.**
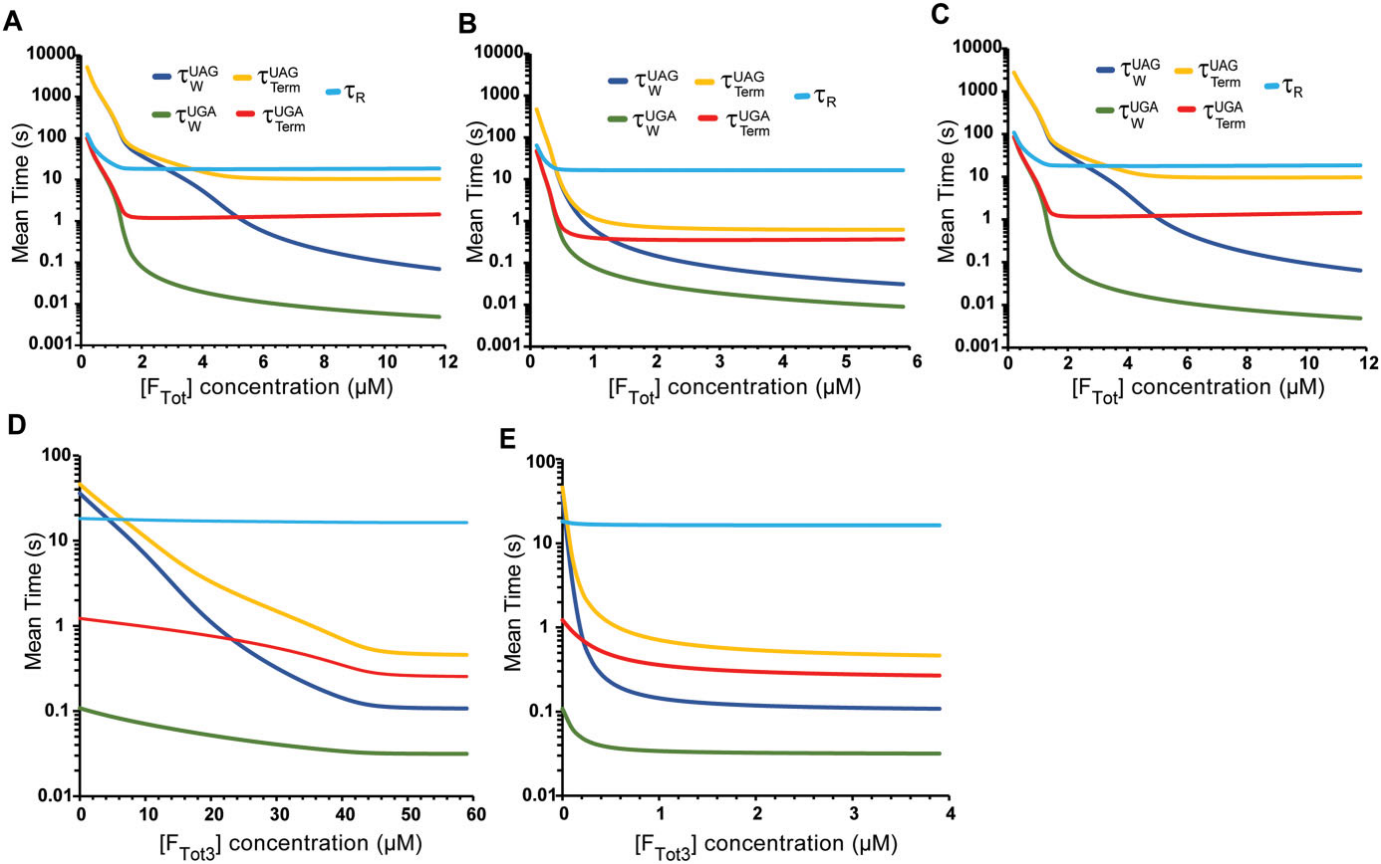
Cellular termination times at varying class-I and -II RF concentrations. (A–C) Mean times for ribosomal termination complexes (y-axes) as functions of total class-I RF concentration, [F_Tot_], with 12% RF1 and 88% RF2 (x-axes) in the absence of RF3 (**A**) or in the presence of RF3 ([F_Tot3_] = 1 μM) with the spontaneous GDP-to-GTP exchange on RF3 in solution assisted by the fast ribosome dependent GDP-to-GTP exchange on RF3 (**B**) or without the fast ribosome assisted GDP-to-GTP exchange (**C**). Waiting (duration) time for complex R_A_ at UAG codon (dark blue,$\tau _W^{UAG}$), UGA codon (green,$\tau _W^{UGA}$); total termination time (total duration time) for all termination complexes from R_A_ to R_P_ at UAG codon (brown,$\tau _{Term}^{UAG}$), UGA codon (red,$\tau _{Term}^{UGA}$); total ribosome cycle time (light blue,${\tau }_R$). Under all simulated conditions waiting and termination times on UGA and UAA (both mainly read by RF2) are virtually identical. (D, E) Waiting and termination times (y-axes) as functions of total RF3 concentration ([F_Tot3_]) at fixed total class-I RF concentration [F_Tot_] = 1.8 μM (12% RF1 and 82% RF2) in absence (**D**) or presence (**E**) of the ribosome-dependent GDP-to-GTP exchange on RF3.

To illustrate system behavior we simulated titration of total class-I RF concentration (on plus off the ribosome), [F_Tot_] = [RF1_Tot_] + [RF2_Tot_], from near zero to 12 μM with ratio [RF2_Tot_] / [RF1_Tot_] fixed at 7 as *in vivo* (19,47). Increasing [F_Tot_] in the absence of RF3 (Figure 6A) decreases waiting times of pre-termination ribosome R_A_ (Figure 5) with UAG (dark blue, $\tau _W^{UAG}$) or UGA/UAA (green, $\tau _W^{UGA}$) stop codon in the A site for the arrival of RF1 or RF2 from about 1000 s or 100 s, respectively, to sub-second values. For [F_Tot_] well below 2μM all waiting times are long enough to dominate the total ribosomal cycling time ${\tau }_R$ (light blue) that includes the times for initiation, elongation, termination and recycling back to initiation. We note here that relative variations in the average ribosomal cycling time ${\tau }_R$ translate directly into relative variations of the cell doubling time (Materials and Methods: Eq. 15). Thus, long ${\tau }_R$ times due to low free [RF1] and [RF2] levels (Figure 6) result in long doubling times of cell populations. The doubling times may increase further due to indirect effects of ribosome pausing at stop codons, like read through by aminoacyl-tRNAs, leading to toxic proteins. The total termination time, given by the sum of lifetimes for R_A_, R_A_·RF_i_, R_P_·RF_i_ and R_P_ states (Figure 5) decreases from 100 s for UGA stop codon (red, $\tau _{Term}^{UGA}$) at the smallest [F_Tot_] to a minimum slightly above 1 s at an [F_Tot_] value about 1.8 μM. At this value of [F_Tot_], the free concentration of RF2 is high enough to reduce the waiting time $\tau _W^{UGA}$ to sub-second range so that the termination time $\tau _{Term}^{UGA}$ is dominated by the time of spontaneous RF2 dissociation from post termination complex R_P_**·**RF_i_. Further increase of [F_Tot_] to 12 μM increases the frequency of RF2 re-binding to ribosomal complex R_P_ causing $\tau _{Term}^{UGA}$ to increase gradually to a value slightly above 3 s. We note that termination on UAA in RF3 absence is mainly done by RF2 so that $\tau _W^{UAA}$ and $\tau _{Term}^{UAA}$ dependences on [F_Tot_] not shown in Figure 6 are very similar to those for $\tau _W^{UGA}$ and $\tau _{Term}^{UGA}$, respectively. The total termination time for UAG stop codons (brown,$\tau _{Term}^{UAG}$) decreases from about 1000 s at the smallest [F_Tot_] toward a plateau value of 10 s, corresponding to the time for spontaneous RF1 dissociation from the post-termination complex as free [RF1] increases in the high-[F_Tot_] range. Finally, the average ribosome cycle time (${\tau }_R$) decreases from about 100 s to 18 s with increasing [F_Tot_] (light blue line).

To summarize, there are three distinct phases in the class-I RF titrations without RF3: a first, ‘RF-starvation phase’ at small [F_Tot_] values where the lifetime of ribosomal state R_A_ (‘waiting’ time) dominates the total termination time (Figure 6A); a second, ‘RF dissociation phase’ at intermediate [F_Tot_] values close to *in vivo* RF1 plus RF2 total concentration of about 2 μM (Figure 6A); and a third phase of ‘RF-inhibited ribosomal recycling’ due to class-I RF re-binding at high [F_Tot_] values (Figure 6A, red).

We note also that at about 2 μM [F_Tot_] the average ribosome cycle time ${\tau }_R$ is already about 18 s and less than 10% longer than${\tau }_R$ obtained assuming instantaneous release of RF1 and RF2 from the post-termination ribosome and no rebinding to the post termination state R_P_. This explains why at [F_Tot_] close to or above its physiological value of about 2 μM, RF3 deletion has but a marginal effect on *E. coli* growth (19,43). Interestingly, our modeling also shows that without RF3, RF2 completely dominates termination on UAA codons (with more than 99% of UAA terminations done by RF2) despite an about 3–5-fold higher efficiency (*k*_cat_/*K*_m_) of RF1 over RF2 in UAA decoding (12,48)

We next modeled [F_Tot_] class-I RF titrations with RF3 total concentration fixed at 1 μM assuming that both pathways of guanine nucleotide exchange on RF3: the spontaneous (B) and ribosome-stimulated (A), were operational (Figure 6B) (see Supplementary Table S1 for parameter values). Here, increasing [F_Tot_] from near zero to values just above 0.5 μM brings UAG, UAA and UGA codons out of their ‘RF_i_-starvation’ phase (Figure 6A, SI) and further increase of [F_Tot_] reduces termination times for UGA/UAA ($\tau _{Term}^{UGA}$) and UAG ($\tau _{Term}^{UAG}$) to values below 0.5 s. We note that in these titrations more than 95% of RF3-dependent termination events occur through pathway A, i.e. through ribosome assisted GDP-to-GTP exchange on RF3 (Figure 5).

Spontaneous GDP-to-GTP exchange on RF3 off the ribosome is rate limited by dissociation of GDP from RF3·GDP with rate constant 0.03 s^−1^ (23) (Supplementary Table S1). Assuming that only such spontaneous regeneration of RF3·GDP to RF3·GTP is operational, the [F_Tot_] class-I RF titration curves in the presence (Figure 6C) and absence (Figure 6A) of 1 μM total RF3 are virtually indistinguishable, implying negligible intracellular RF3 activity in termination without ribosome-enhanced guanine nucleotide exchange. Indeed, the modeling shows that at the physiologically relevant 1.8 μM [F_Tot_] level only about 2.5% of termination events are catalyzed by RF3**·**GTP that is spontaneously generated from free RF3**·**GDP in solution (see also Discussion).

To illustrate further how variation in total RF3 concentration ([F_Tot3_]) affects termination efficiency, we kept [F_Tot_] of class-I RF constant at 1.8 μM, similar to that estimated *in vivo* (13), while [F_Tot3_] was either titrated from zero to 50 μM assuming only spontaneous GDP-to-GTP exchange on free RF3 in solution (Figure 6D), or from zero to 6 μM assuming both spontaneous and ribosome-enhanced nucleotide exchange on RF3 operational (Figure 6E). In the former case, the mean waiting time $\tau _W^{UAG}$(bark blue) and total time $\tau _{Term}^{UAG}$ (yellow) of termination on UAG codons by RF1 decrease gradually from about 36 and 46 s, respectively, to sub-second values as [F_Tot3_] increases toward 50 μM. With both spontaneous and ribosome-enhanced nucleotide exchange operational, $\tau _W^{UAG}$and $\tau _{Term}^{UAG}$ decrease sharply to sub-second values as [F_Tot3_] increases from 0 to 0.8 μM, i.e. to the total RF3 concentration estimated *in vivo* (13). This means that in fast growing cells the spontaneous GDP-to-GTP exchange on free RF3 alone cannot provide the required rate of RF3·GTP regeneration at physiologically relevant RF3 concentrations. In other words, without the contribution from ribosome-stimulated RF3·GTP regeneration the enhancement of the termination efficiency by RF3 would require a non-physiologically high total intracellular RF3 concentration in the 0.1 mM range (Figure 6D).

## DISCUSSION

Applying real-time single-molecule detection of ribosome conformation and occupancy of class-I RFs RF1/RF2 and class-II RF RF3 during termination, we reveal complex RF3 sampling dynamics of non-productive binding events. These occur on a given terminating ribosome prior to the productive RF3 binding event that greatly increases the rate of intersubunit rotation in a GTP-dependent manner. This rotation destabilizes class-I RF binding and induces rapid class-I RF dissociation followed by GTP hydrolysis and dissociation of RF3 in the GDP form. From direct observation of RF1 and RF3 dissociation events after ribosome rotation we established that RF1 dissociation occurs first and is followed by RF3 dissociation. We also observed a small but significant delay of class-I RF dissociation from the fully rotated ribosome in the presence, but not absence of ribosome bound RF3 (Figure 3B). The reason for this delay is, we suggest, that the presence of RF3 not only reduces the free energy barrier between the un-rotated and rotated ribosome, but also slightly stabilizes class-I RF binding to the fully rotated ribosome. Experiments with the non-hydrolysable GTP analogue GDPCP show rapid intersubunit rotation followed by rapid release of RF1 and a 270-fold increase in RF3 occupancy time on the rotated ribosome after RF1 dissociation. This shows that the GTPase activity of RF3 has little influence on intersubunit rotation and RF1 dissociation times but is crucial for subsequent release of RF3 from the post-termination ribosome.

Previously reported data on RF1 and RF3 dissociation kinetics have led to different conclusions on the order of dissociation events during termination (24,30). In contrast to these previous studies our approach clarifies the order of the events directly, through simultaneous tracking of ribosome occupancy by RF1 and RF3 and correlation of their binding and dissociation events with ribosomal intersubunit rotations. Tracking ribosome conformations in our assay system has revealed two different modes of RF3 binding to the non-rotated ribosome: a sampling mode, where a bound RF3 can rapidly dissociate in a GTP hydrolysis-independent fashion; and a productive mode, where RF3 binding results in ribosome rotation followed by dissociation of RF1 and RF3 in a GTP hydrolysis-dependent fashion (Figure 3). The existence of the sampling mode provides an alternative explanation for the fast GTP hydrolysis-independent RF3 dissociation events that were previously observed (24).

Dissociation kinetics for the non-productive RF3 sampling revealed that intersubunit rotation is initially blocked for post-termination ribosomes with a ‘first-bound’ class-I RF in the A site both in absence and presence of RF3 (Figure 2, Supplementary Figure S2). Intersubunit rotation competence at a relatively low, 20°C temperature, is gained in an RF3-independent activation step over a large, enthalpy-dominated standard free energy barrier (Figure 2F). Activation is impaired by inhibitors, like the antimicrobial drug Api137, which preclude both spontaneous and RF3-accelerated ribosome intersubunit rotation and leads to perpetual cycles of RF3 sampling (Supplementary Figure S4). Api137 binds in the peptidyl-transferase center (PTC) where it stabilizes the hydrogen-bonding interactions between RF1 and P-site tRNA (22), thereby impairing tRNA flexibility that is required to allow for ribosome intersubunit rotation (29).

What, then, is the nature of the high-enthalpy activation step preceding intersubunit rotation for post-termination ribosomes with a ‘first-bound’ class-I RF in the A site and why is the blockage absent (Supplementary Figure S2D) for a ‘rebound’ class-I RF? We note that at 20°C the activation step (min range) is much slower than the rapid steps known to follow first class-I RF binding to the A site (ms range), including conformational change of the class-I RF (7), hydrolysis of ester bond in peptidyl-tRNA and dissociation of nascent peptide from the ribosome (8). Although the duration time for these initial steps is far too short for any one of them to be identified as the slow activation step, it is conceivable that ester bond hydrolysis in P-site tRNA by a class-I RF could allow for a subsequent slow conformational change that activates the ribosome for intersubunit rotation. A candidate for such an activation step could include movement of originally P-site bound tRNA to a transient position between P and E site as observed in cryo-EM studies of RF1 recycling (29) as well as a structural change of the GGQ loop of class-I RF to beta hairpin (49). Once activated, the ribosome would allow for intersubunit rotation with a rate constant greatly increased by introduction of ribosome bound RF3·GTP (Figure 2G). Peptide-lacking, class-I RF rebound ribosomes would, by hypothesis, already be activated for intersubunit rotation upon RF binding (Supplementary Figure S2D), as shown here.

We observed RF3 sampling in the presence of GTP, GDP, GDPCP and a fifty-fifty mixture of GTP and GDP free in solution (Figure 3–Figure 4). RF3 occupancy times for these sampling events reveal heterogeneous distributions with up to three relaxation times, which suggest the existence of at least three sampling states of ribosome -bound RF3, with kinetic properties modulated by guanine nucleotide identities and mixtures. Termination with RF1 and RF3 at 20°C in the presence of only GDP produces a rapidly-dissociating (>4 s^−1^) RF3 sampling state whereas the presence of only GTP or GDPCP yields a state with intermediate dissociation rate constant (1–2 s^−1^) (Figure 4F, G). Both sub-states described above in the presence of GDP or GTP/GDPCP with corresponding kinetics were present at equal amplitude when terminating in the presence of a 50:50 concentration mixture of GDP and GTP. Progression to a productive RF3 event requires a GTP-dependent mechanism, as increasing fraction of GTP in a GTP:GDP mixture accelerates the RF3-mediated ribosome rotation (Supplementary Figure S5B). With all guanine nucleotides, a very slowly dissociating (<0.4 s^−1^) RF3 state was observed, suggesting a ribosome-bound RF3 state that is independent of type of guanine nucleotide. We speculate that this high-affinity state represents an apo-state of RF3 as identified in previous biochemical studies and found to have high affinity for the post-termination RF1-ribosome complex (50). The observations of RF3·GDP and possibly apo-RF3 states on the ribosome and the GTP-dependent acceleration of intersubunit rotation, agree with previously proposed (23) models of termination where ribosome-dependent exchange of initially-bound RF3·GDP to RF3·GTP via an apo-RF3 intermediate catalyzes the ribosome rotation and subsequent recycling of release factors.

The present global modelling of cellular protein synthesis shows that the previously demonstrated rapid GDP to GTP exchange on ribosome bound RF3 (Pathway A in Figure 5) (12,16,23,25,26) rather than ‘spontaneous’ exchange on free RF3 in the cytoplasm (Pathway B in Figure 5) (16) is the dominant pathway for RF3**·**GTP regeneration in the growing living cell. We found the *in vivo* concentration ratio [RF3**·**GTP]/[RF3**·**GDP] to be determined by the supply and demand flows for RF3**·**GTP rather than by its equilibrium value, in contrast to a previous claim (16). To provide a simple illustration of the logic behind this conclusion, we assume an intracellular concentration of elongating ribosomes of 20 μM (13), a peptide elongation rate per codon of 20 s^−1^ (46) and a mean protein size of 300 AAs implying that 1.33 μM such protein molecules are produced in the cell per second, This value, 1.33 μM·s^−1,^ correspond to a molar termination demand flow for RF3**·**GTP. In the ‘spontaneous’ exchange case, a total RF3 concentration *in vivo* of 1 μM (13) and a rate constant for GDP dissociation from free RF3 of 0.03 s^−1^ (23) leads to an upper limit for the molar supply RF3·GTP flow of 0.03 μM·s^−1^, achievable only at an infinite GTP to GDP concentration ratio (see SI). In cases of more realistic cellular free GTP to GDP concentration ratios (27), the molar supply flow of RF3**·**GTP regenerated from RF3·GDP in solution would be even smaller (see SI). From this follows that ‘spontaneous’ GTP to GDP exchange on RF3 in solution (Figure 5, pathway B) can contribute to the rate of termination in less than 2.5% of actually occurring termination events, resulting in a termination scenario reminiscent of complete RF3 deficiency (Figure 5, pathway C, Figure 6C). To match the cellular termination demand and supply flows for RF3·GTP when supply is based on ‘spontaneous’ RF3·GTP regeneration would require very high intracellular RF3 concentrations in the 0.1 mM range (Figure 6D). Therefore, at physiological RF3 concentrations below 1 μM (13) the previously discovered mechanism of ribosome catalyzed GDP to GTP exchange for RF3 (12,16,23,25,26), (depicted as pathway A in Figure 5), is required for RF3 enhanced rate of translation termination in the growing bacterial cell.

Given RF3’s role as an enhancer of the rate of termination, it might seem surprising that RF3 deficiency causes but small growth defects in a set of studied *E. coli* strains (43). These *in vivo* results are, however, fully in line with our modeling predictions. The reason is that the mean time of spontaneous RF2 dissociation from the post-termination ribosome is only about 1 s in the absence of RF3 (24) (Figure 5, pathway C), a time much smaller than the average ribosome translation cycle time ${\tau }_R$ (Materials and Methods: Eq. 13) of about 17 s in the presence of RF3. Since RF2 is the major class-I RF and the main reader of UGA and UAA codons RF3 deletion is expected to increase ${\tau }_R$ by only about 1 s, i.e. by <7% and, hence, increase the cell doubling time T_D_ by <7% too (Materials and Methods: Eq. 15). This conclusion is not much affected by the role played by RF1, although its mean spontaneous dissociation time, 10s, is considerably longer than that of RF2 (17,24). This is because RF1 is a minor class-I RF and only a small fraction of ribosomes translate mRNAs with the UAG stop codon with RF1 as its only cognate reader.

Since many bacterial strains seem to lack RF3 (44), an interesting question is whether *E. coli* strains are in the process of ‘phasing out’ RF3 by compensating for loss in its termination activity by evolving increased rate constants for un-catalyzed RF1 and RF2 dissociation from the post-termination ribosome (pathway ‘C’ in Figure 5).

In summary, bulk and single-molecule kinetics combined with *in vivo* modeling support a guanine-nucleotide exchange mechanism, whereby the post-termination ribosome accelerates RF3·GDP to GTP exchange followed by ribosomal subunit rotation, rapid RF1 and subsequent rapid GTPase dependent RF3 departure.

## DATA AVAILABILITY

Data available upon request.

The following MATLAB data analysis scripts are deposited as supporting files:

- A: Initial automated selection of ZMWs based on fluorescence
  - Saved as A_FilterZMW_Fluorescence.m
- B: Manual selection of ZMWs singly-loaded with productive ribosome complexes
  - Saved as B_SelectZMWtraces.m
- C: Conformational and compositional state assignment to generate a hidden Markov model (HMM) matrix
  - Saved as C_AssignStates.m
- D: Collection of data analysis scripts used to extract temporal data from the HMM matrix compressed into a zip file
  - Saved as D_TemporalAnalysisScripts.rar

## Supplementary Material

gkad286_Supplemental_Files

## Notes

Present address: Arjun Prabhakar, Pacific Biosciences, Inc., Menlo Park, CA 94025, USA.
