# Supplementary material for: Dynamics of release factor recycling during translation termination in bacteria
Source: Nucleic Acids Res. 2023 Apr 27;51(11):5774–90. doi: 10.1093/nar/gkad286 (PMC10287982; doi:10.1093/nar/gkad286)
Supplement: gkad286_Supplemental_Files [file gkad286_supplemental_files.zip › Supp_Information_SecondRevision_Final.pdf]

Supplementary Information

Dynamics of release factor recycling during translation termination in bacteria

Arjun Prabhakar, Michael Y. Pavlov, Jingji Zhang, Gabriele Indrisiunaite, Jinfan Wang, Michael R. Lawson, Måns Ehrenberg, Joseph D. Puglisi

This file contains:

Part A: Supplementary Figures (S1-S7) and Supplementary Table S1

Part B: Supplementary Text (SI Text)

Part A:

Supplementary Figures S1-S7

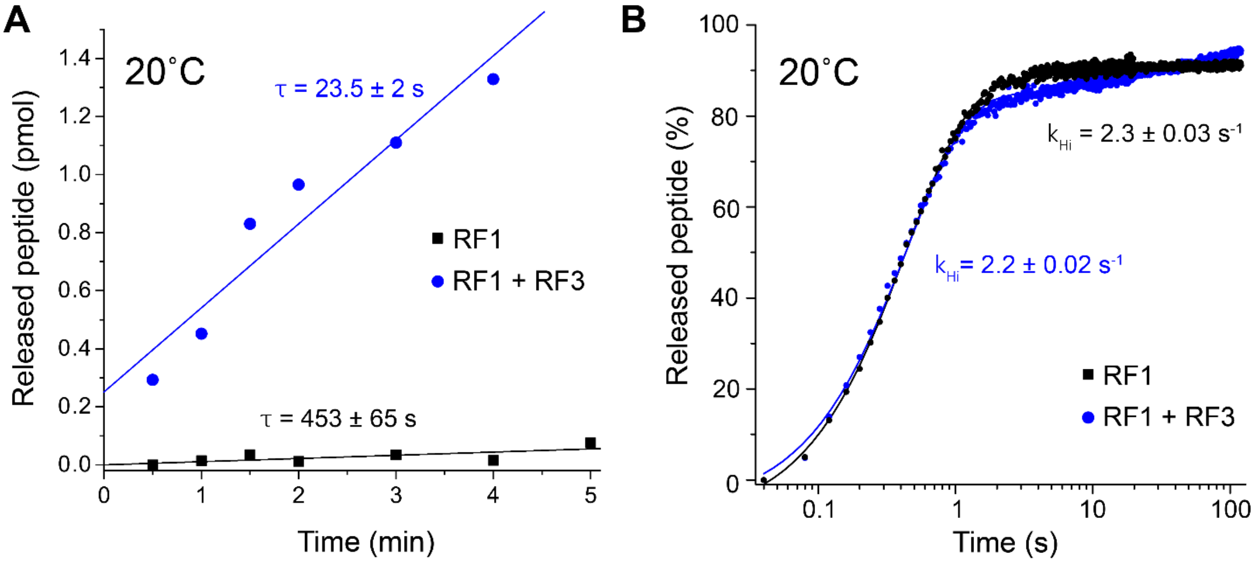

Figure S1. Bulk kinetics measurements of peptide release by RF1

(A) Time courses of RF1 (0.1 pmol) recycling on ribosome complex (RC, 5 pmol) at 20°C with (blue dots) or without (black squares) RF3 (5 pmol). RC had UAA programmed A site and ^{3}H-fMet-Phe-Phe-tRNA^{Phe} in P site. Recycling times (τ) given as averages ± s.d.

(B) Time courses of RF1-catalyzed ester bond hydrolysis at 20°C in coumarin-labeled 3H-Met-Phe-Phe-tRNA^{Phe} in P site of UAA programmed RC. At time zero in a stopped flow instrument solutions of RC (0.05 µM) and RF1 (3.4 µM) were rapidly mixed in the absence (black dots) or presence (blue dots) of RF3 (8 µM) for detection of the following coumarin fluorescence change.

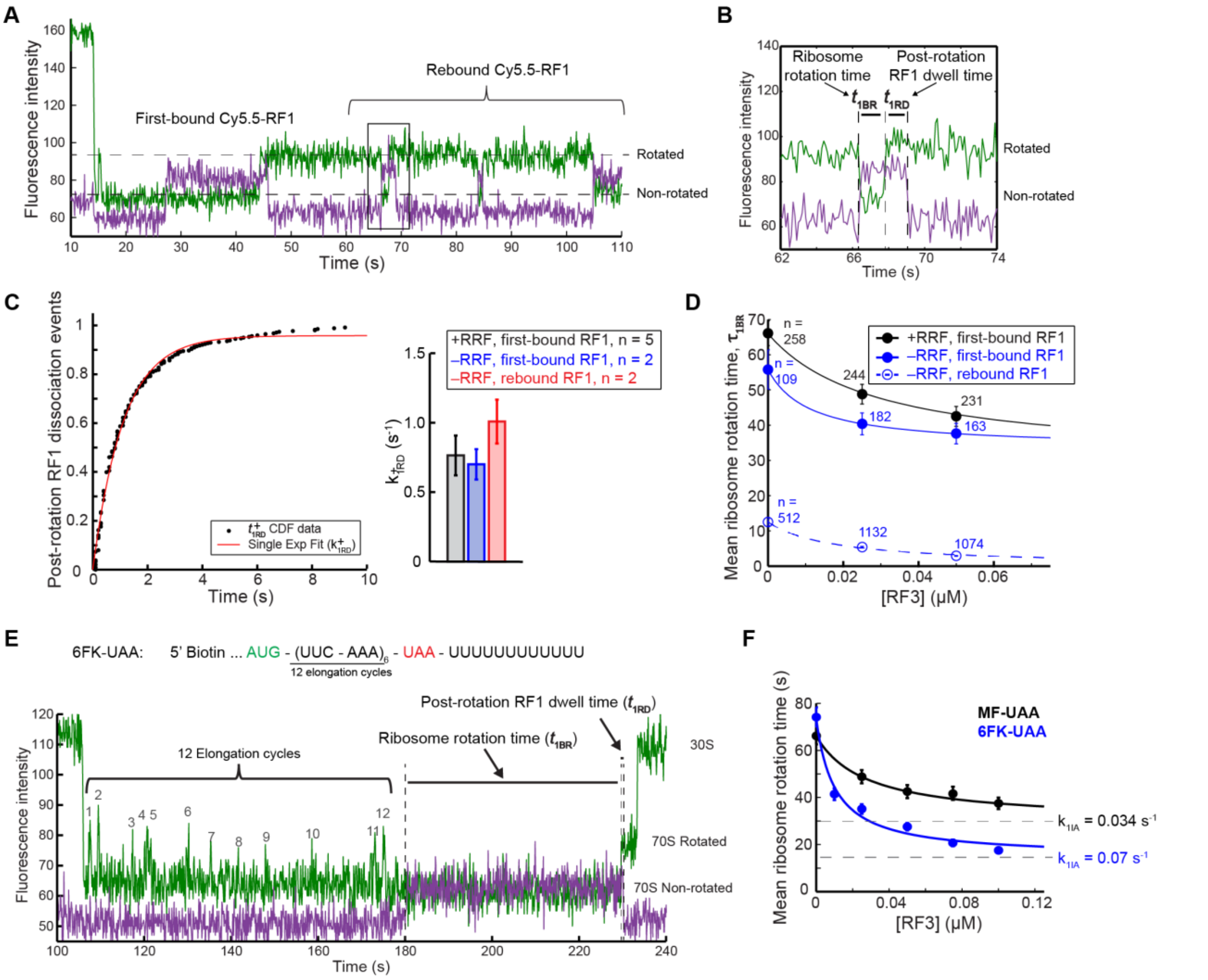

Figure S2. Post-termination recycling of RF1 is gated by a slow [RF3]-independent activation step

(A) Representative trace of ribosome translating MF-UAA mRNA in the presence of TCs, EF-G, Cy5.5-RF1 and RF3. In the absence of RRF, post-termination Cy5.5-RF1 dissociation is followed by multiple cycles of Cy5.5-RF1 rebinding events that are correlated with ribosome transition from rotated to non-rotated state.

(B) Subsection of trace in panel **(A)** defining the ribosome rotation time and post-rotation RF1 dwell time of a rebound Cy5.5-RF1.

(C) Left: Sample cumulative distribution of post-rotation RF1 dwell times conditional on RF3 binding (t_{1RD}^{+}) of rebound RF1 fit to a single exponential function with rate constant k_{1RD}^{+}. Right: Post-rotation RF1 dissociation rate constant (k_{1RD}^{+}) of the first-time bound RF1 and rebound RF1 measured in the presence and absence of RRF. Error bars are defined as SD of multiple rate constants calculated from exponential fits of multiple datasets collected in the presence of RF3.

(D) Mean ribosome rotation time (τ_{1BR}) measured from the first-bound RF1 and rebound RF1 as a function of [RF3], with reported sample size n for each dataset. Error bars are defined as S.E.M.

(E) Representative trace of ribosome translating 6FK-UAA mRNA in the presence of TCs, EF-G, RRF, Cy5.5-RF1 and dark RF3, highlighting the 12 cycles of elongation and RF3-driven ribosome rotation before RF1 dissociation, which defines the ribosome rotation time. Post-rotation RF1 dwell time is defined as the time interval between ribosome rotation and RF1 dissociation.

(F) Mean ribosome rotation times (τ_{1BR}) measured from Cy5.5-RF1 experiments at 20°C with MF-UAA and 6FK-UAA mRNAs as a function of [RF3]. The data were fit to **M&M:** Eq. 7 to determine the rate constant of [RF3]-independent activation (k_{1IA}). Sample sizes for the MF-UAA datapoints are reported in **Figure 2A** and those for 6FK-UAA datapoints in increasing [RF3] order are n = 232, 228, 224, 242, 243, and 230. Error bars are defined as S.E.M.

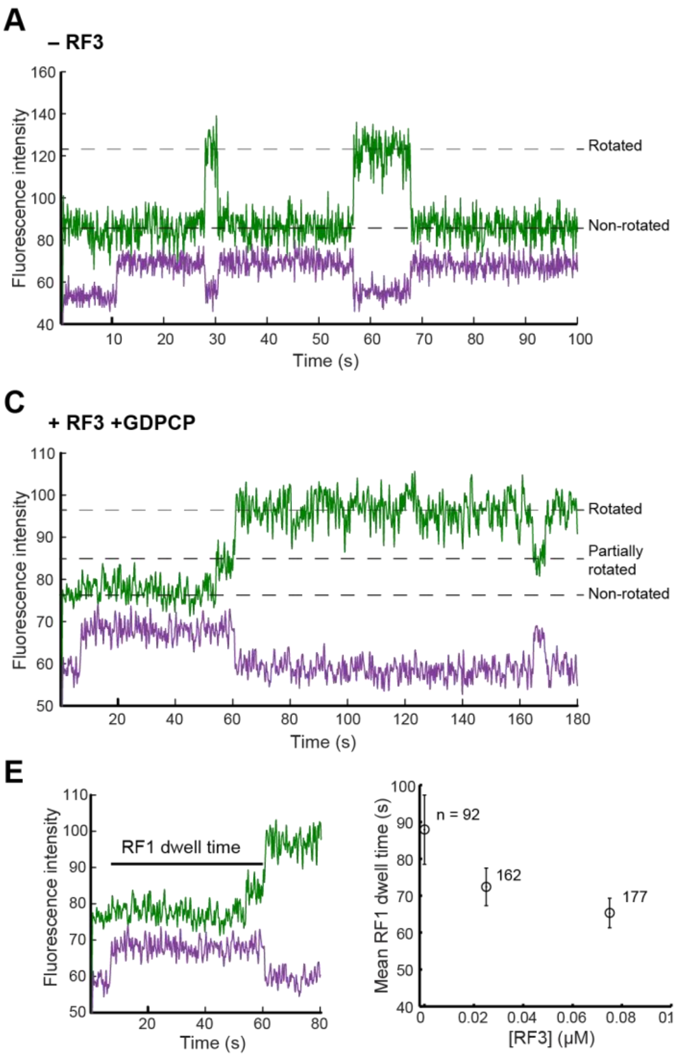

Figure S3. GDPCP introduces a partially rotated state during RF3-driven ribosome rotation

(A) Representative trace of ribosomes terminated by Cy5.5-RF1 with only GDPCP (no RF3) in the absence of RRF and EF-G. Without RRF, the post-termination ribosome undergo Cy5.5-RF1 rebinding events correlated to transition from rotated to non-rotated ribosome conformation, shown also in the presence of GTP in **Supplementary Figure S2A**.

(B) Contour plots of Cy3B intersubunit rotation signal and Cy5.5-RF1 occupancy post-synchronized to timepoints of post-termination ribosome rotation and Cy5.5-RF1 rebinding timepoints.

(C) Representative trace of ribosomes terminated by Cy5.5-RF1 and dark RF3·GDPCP in the absence of RRF and EF-G. RF3·GDPCP introduces a partially rotated intermediate from which a transition into rotated state is correlated to Cy5.5-RF1 dissociation step. Cy5.5-RF1 rebinding afterwards to the ribosome are correlated to transition from rotated to partially rotated ribosome conformation.

(D) Contour plots of Cy3B intersubunit rotation signal and Cy5.5-RF1 occupancy post-synchronized to timepoints of partial rotation and Cy5.5-RF1 rebinding timepoints.

(E) Mean RF1 dwell time of the first RF1 binding event as a function of [RF3]. Error bars are defined as S.E.M.

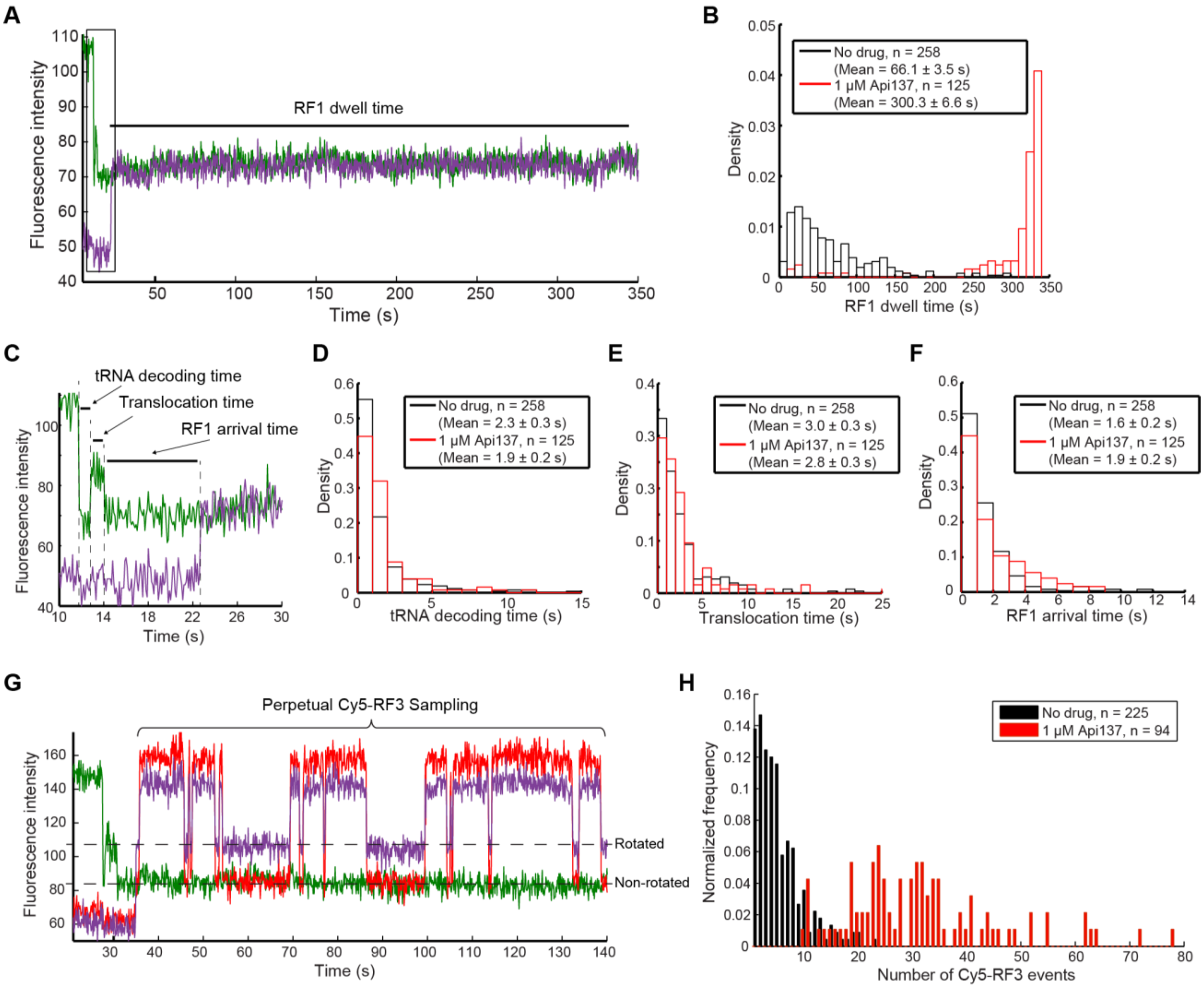

Figure S4. Api137 inhibits intersubunit rotation

(A) Representative trace of ribosome translating MF-UAA mRNA in the presence of GTP, TCs, EF-G, RRF, Cy5.5-RF1 and Api137, with RF1 dwell time defined. Api137 traps Cy5.5-RF1 on the ribosome until the end of the movie (6 min).

(B) Histogram of the distribution of RF1 occupancy times in the absence and presence of 1µM Api137. Errors are defined as S.E.M.

(C) Subsection of trace in panel **(A)** defining the Phe-tRNA^{Phe} decoding time, translocation time, and RF1 arrival time.

(D) Histogram of the distribution of tRNA decoding times in the absence and presence of 1µM Api137. Errors are defined as S.E.M.

(E) Histogram of the distribution of translocation times in the absence and presence of 1µM Api137. Errors are defined as S.E.M.

(F) Histogram of the distribution of RF1 arrival times in the absence and presence of 1µM Api137. Errors are defined as S.E.M.

(G) Representative trace of ribosome translating on MF-UAA mRNA in the presence of TCs, EF-G, F4, Cy5.5-RF1, Cy5-RF3, and Api137. In the presence of Api137, Cy5-RF3 undergoes perpetual cycles of sampling events throughout the entire movie without ribosome rotation.

(H) Frequency distribution of number of Cy5-RF3 events shows that the frequency is dramatically increased in the presence of Api137.

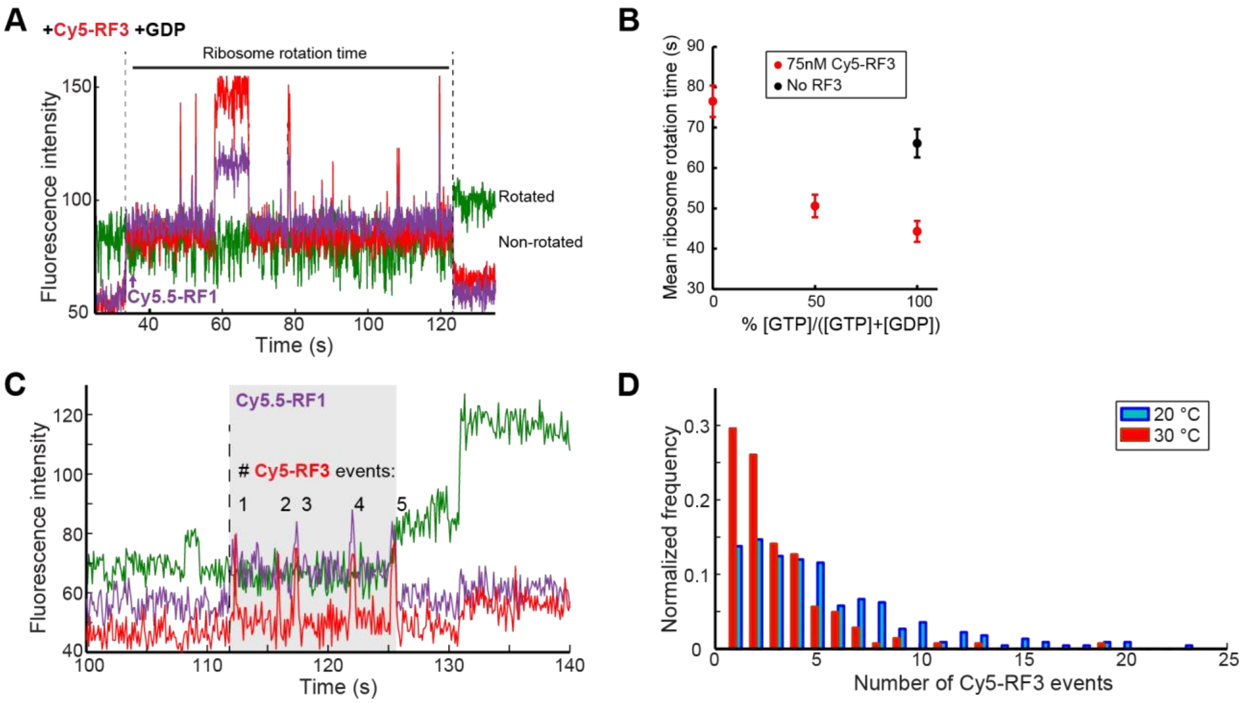

Figure S5. RF3 samples the non-rotated RF1-ribosome in the presence of GTP, GDP, or GDPCP

(A) Representative trace of Cy5-RF3 sampling on post-termination ribosome observed in the presence of GDP. Ribosome rotation time is marked as the dwell time between the arrival of Cy5.5-RF1 and ribosome rotation signal.

(B) Mean ribosome rotation time as a function of the percent of GTP in total mixture of GTP and GDP. Error bars are defined as S.E.M.

(C) Representative trace of ribosome terminating with Cy5.5-RF1, Cy5-RF3, and GTP at 30°C. We quantify the frequency of Cy5-RF3 events by counting the number of Cy5-RF3 signals during occupancy of Cy5.5-RF1.

(D) Normalized frequency distribution of number of Cy5-RF3 events in the presence of GTP shows that the frequency decreases with increase in temperature from 20°C to 30°C.

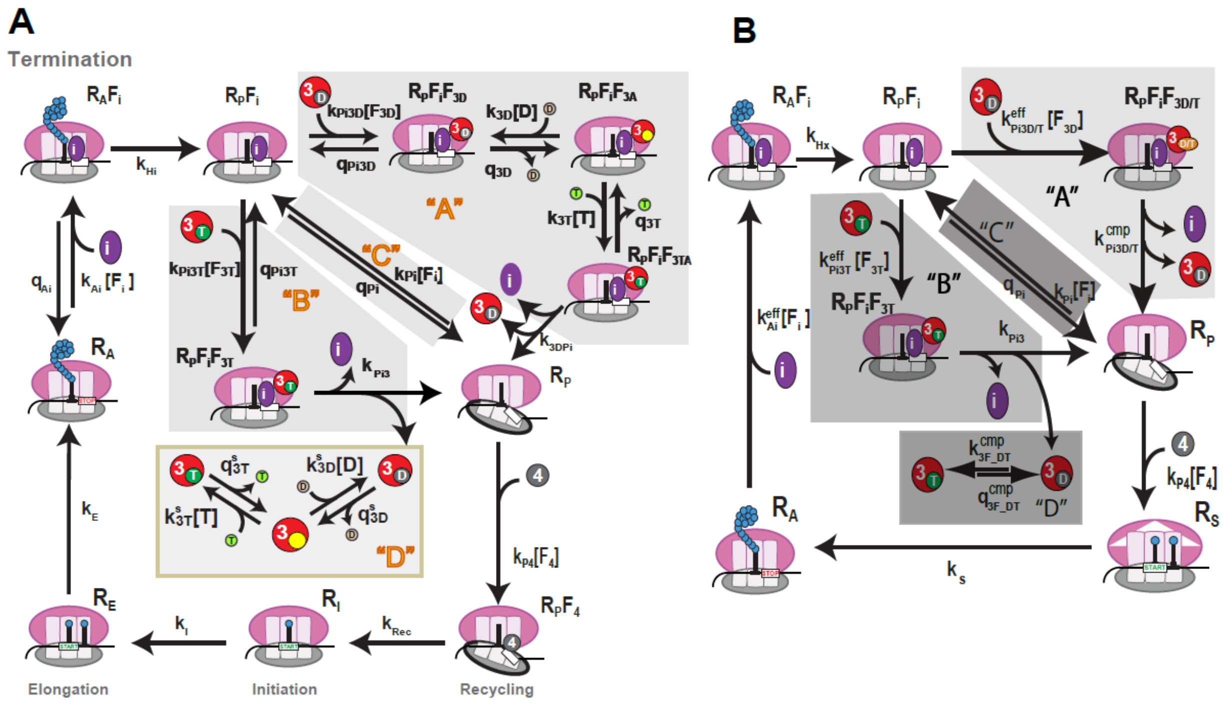

Figure S6. (A) Reproduction of the detailed kinetic scheme in **Figure 5** of the main text in which release and recycling factor notation RF$_i$, RF3·GDP, RF3·GTP, apo-RF3· and RRF are replaced by short-hand notation Fi, F_{3D}, F_{3T}, F_{3A} and F_4, respectively. **(B)** Contracted scheme of protein synthesis with steady state properties identical to those of the high kinetic resolution scheme in (A). We note that complexes R_pF_4, R_I and R_E in (A) are formally replaced here with a virtual complex R_S which steady state concentration is the sum of the steady state concentrations of R_pF_4, R_I and R_E. Further, three complexes $R_PF_iF_{3D}$ (R_P·RF$_i$· RF3·GDP), $R_PF_iF_{3A}$ (R_P·RF$_i$· RF3·) and $R_PF_iF_{3AT}$ (R_P·RF$_i$· RF3·GDP) on the pathway "A" in scheme (A) are formally replaced here by a virtual complex $R_PF_iF_{3D/T}$ which steady state concentration is the sum of the steady state concentrations of $R_PF_iF_{3D}$, $R_PF_iF_{3A}$ and $R_PF_iF_{3AT}$.

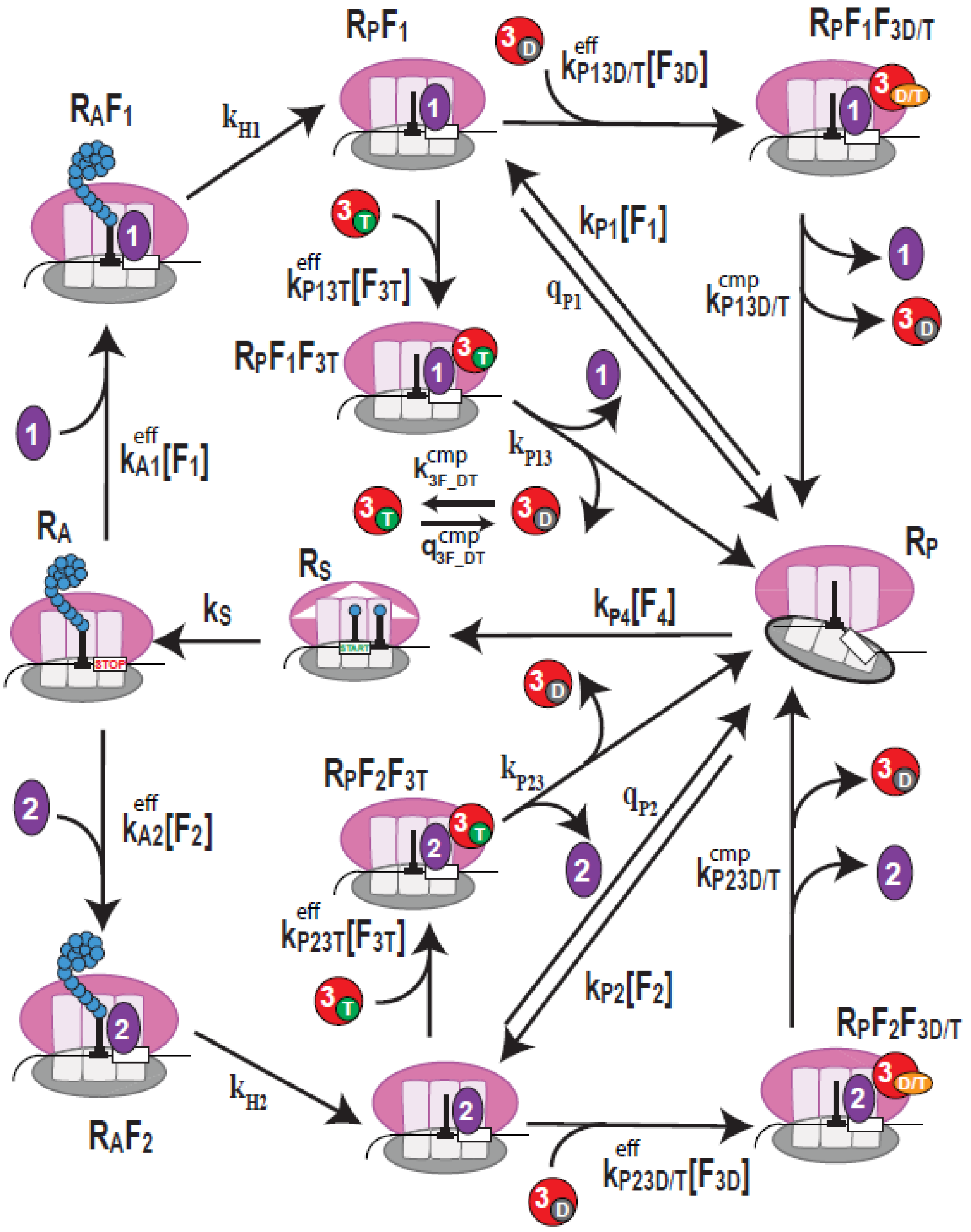

Figure S7. Contracted scheme for ribosomes translating UAA containing mRNAs.

Supplementary Table S1

Table S1. Kinetic parameters used in model calculations

Description	Rate constant	Value at 37°C
Effective RF1 association rate constant to pre-termination complex R_A (=k_{cat}/K_M parameter of F_1 termination)	k_{A1}^{eff}	60 μ$M^{-1}s^{-1}$ (Zavialov et al, 2002)
kcat parameter for termination by F_1	k_{H1}	17 s^{-1} (Indrisiunaite et al, 2015)
Spontaneous RF1 dissociation rate constant from post-termination complex R_pF_1	q_{P1}	0.1 s^{-1} (Pavlov et al, 1997)
RF1 re-association rate constant to post-termination complex R_P	k_{P1}	60 μ$M^{-1}s^{-1}$ (*)
Effective RF2 association rate to pre-termination complex R_A (=k_{cat}/K_M parameter of F_2termination)	k_{A2}^{eff}	23 μ$M^{-1}s^{-1}$ (Zavialov et al, 2002)
kcat parameter for termination by F_2	k_{H2}	22 s^{-1} (Indrisiunaite et al, 2015)
Spontaneous RF2 dissociation rate constant from post-termination complex R_pF_2	q_{P2}	1 s^{-1} (Pavlov et al, 1997)
RF2 re-association rate constant to post-termination complex R_P	k_{P2}	23 μ$M^{-1}s^{-1}$ (*)
Effective association rate constant of RF3:GDP to post-termination complex R_pF_1 to form a virtual complex $R_pF_1F_{3D/T}$	$k_{P13D/T}^{eff}$	5 μ$M^{-1}s^{-1}$ (**)
Compounded rate constant for disassembly of virtual $R_pF_1F_{3D/T}$ complex that results in ejection of RF1 and RF3:GDP from the ribosome	$k_{P13D/T}^{cmp}$	2 s^{-1} (***)
Effective association rate constant of RF3:GDP to post-termination complex R_pF_2 to form a virtual complex $R_pF_2F_{3D/T}$	$k_{P23D/T}^{eff}$	5 μ$M^{-1}s^{-1}$ (**)
Compounded rate constant for disassembly of the virtual $R_pF_2F_{3D/T}$ complex that results in ejection of RF2 and RF3:GDP from the ribosome	$k_{P23D/T}^{cmp}$	8 s^{-1} (***)
Effective association rate constant of RF3:GTP to post-termination complex R_pF_1 to form complex $R_pF_1F_{3T}$	k_{P13T}^{eff}	6 μ$M^{-1}s^{-1}$ (**)
k_{cat} parameter for GTP-dependent $R_pF_1F_3$ disassembly that results in ejection of RF1 and RF3:GDP from the ribosome	k_{P13}	2 s^{-1} (***)
Effective association rate constant of RF3:GTP to post-termination complex $R_pF_2$to form complex $R_pF_2F_{3T}$	k_{P23T}^{eff}	6 μ$M^{-1}s^{-1}$ (**)
k_{cat} parameter for GTP-dependent $R_pF_2F_3$ disassembly that results in ejection of RF2 and RF3:GDP from the ribosome	k_{P23}	8 s^{-1} (***)
Upper limit for the rate of GDP to GTP exchange on RF3 is solution	$k_{3F\_DT}^{cmp}$	0.03 s^{-1} (Zavialov et al, 2001)
Association rate constant for ribosome recycling factor (RRF) binding to the ribosome with the P-site de-acylated tRNA	k_{F4}	15 μ$M^{-1}s^{-1}$ (Borg et al, 2016)
kcat of the recycling process at optimal EF-G concentration	k_{Rec}	5 s^{-1} (Borg et al, 2016)
Initiation rate constant	k_I	1 s^{-1} (Zorzet et al, 2010)
Elongation rate constant for the synthesis of a 300 aa protein assuming a 20 aa/s ribosomal elongation rate	k_E	0.067 s^{-1}

(*) The rebinding association rate constant of F_i to RP complex was assumed to be equal to k_{Ai}^{eff} at 37°C.

(**) Roughly extrapolated from k_{3on} values in **Table 2** assuming a 2.5 fold increase due to the temperature increase from 20° to 37^oC.

(***) Roughly extrapolated from k_{iRD}^{+} values in **Table 2** assuming their 2.5 fold increase due to temperature increase from 20° to 37^oC; the value of k_{iRD}^{-} (**Table 2**) was assumed to increase further about 20 fold as the temperature increased from 30° to 37^oC; the value of k_{31D} (**Table 2**) was assumed to increase by factor of 3 making the contributions of both k_{iRD}^{-} and k_{31D} (see the scheme in **Figure 2G**) into the k_{cat} value of RF3 dependent disassembly of $R_pF_iF_3$ at 37^oC negligible and dominated by k_{iRD}^{+}

Part B: Supplementary Text

RF3 sampling of non-rotated ribosome state and nucleotide-exchange mechanism

In the single-molecule experiment where we tracked the sampling of a non-rotated RF1/2-bound ribosome by Cy5-RF3 in the presence of only GDP, RF3 dissociation was biphasic with a dominant (84% amplitude) rapid (4.6 s^{-1}) and a minor (16% amplitude) slow (0.25 s^{-1}) phase (**Figure 4F**). This suggests that RF3 in the GDP form (RF3·GDP) entered the ribosome forming an R_P·RFi·RF3·GDP complex from which GDP dissociated leading to a comparatively stable R_P·RFi·RF3· complex with ribosome-bound apo-RF3 (RF3·) (**Figure 5**, pathway A). By hypothesis, with high probability, re-formation of R_P·RFi·RF3·GDP by GDP binding to R_P·RFi·RF3· lead to rapid dissociation of RF3·GDP from the ribosome (fast phase). Alternatively, with low probability, R_P·RFi·RF3· became a ribosomal complex with stabilized binding of RF3· and destabilized GDP binding to ribosome bound RF3· (slow phase). At equal concentrations of GTP and GDP there is a fast phase (42%) and a slow phase (13%) similar to those in the only GDP case. There is also an intermediate relaxation rate (1.5 s^{-1}) phase (45%), with similar rate as the fast phase in the GTP-only case (**Figure 4F**).

Prior affinity measurements suggest that GDP has higher affinity than GTP to RF3 (Peske et al, 2014; Zavialov et al, 2001). Furthermore, the more conservative estimate of the affinity difference between the nucleotides from Peske et al. (2014) is likely amplified at 20°C vs 37°C. From these considerations we suggest that in the experiment with equal solution concentrations of GDP and GTP, RF3 primarily entered the post-termination complex in the GDP-bound form, which lead to the same ribosome-bound apo-RF3· intermediates as in the GDP-only and GTP-only cases. At equal GDP and GTP concentrations there was, by hypothesis, 50% probability that either GDP or GTP first bound to ribosome bound apo-RF3·, resulting in rapid RF3 release by GDP rebinding, as in the case of 100% GDP in solution, or slower release by GTP binding as in the case of 100% GTP in solution. In a rare number of cases apo-RF3 reached a deeper state with high ribosome affinity from which it returned to free state by GDP binding and to the ribosome rotation and class-I RF release by GTP binding (RF3 productive binding). This scenario accounts in a simple way for the three dissociation phases that characterize RF3 association to and dissociation from the class-I RF bound ribosome in a fifty-fifty solution mixture of GDP and GTP (**Figure 4F**).

Quantitative modeling of RF3-assisted termination of protein synthesis in living cells

Here we demonstrate that the detailed scheme of protein synthesis in Figure 5 of the main text and, hence, the equivalent scheme shown in Supplementary Figure S6A in which the notations for different complexes are shortened, can be contracted to a much simpler scheme shown in Supplementary Figure S6B that has identical steady state properties and the same termination flows as the schemes in Figure 5 or Supplementary Figure S6A. Main advantages of the contracted scheme in Supplementary Figure S6B is that it has fewer ribosomal complexes and employs compounded rather than elemental rate constants in pertinent cases. Estimates of such compounded rate constants are much easier to obtain from kinetic experiments than elemental rate constants used in the schemes in Figure 5 or Supplementary Figure S6A.

Three termination pathways in *E. coli* bacteria

We note first that Supplementary Figure S6A reproduces Figure 5 of the main text in all but notations for release factors and their ribosome complexes. These shortened notations are convenient for describing the scheme dynamics by differential equation (see below).
Like Supplementary Figure S7A, Supplementary Figure S7B shows that RF3 (F_3) is involved in two of the three termination pathways of *E. coli* bacteria.

In the first pathway **(A)** in Supplementary Figure S6B R_PF_i (R_P·RF_i) binds F_{3D} (RF3·GDP) with compounded rate constant $k^{eff}_{Pi3D/T}$ (k_{cat}/K_m parameter) to form a virtual complex $R_PF_iF_{3D/T}$, composed of three complexes $R_PF_iF_{3D}$ (R_P·RF_i· RF3·GDP), $R_PF_iF_{3A}$ (R_P·RF_i· RF3·) and $R_PF_iF_{3AT}$ (R_P·RF_i· RF3·GTP) depicted in Supplementary Figure S6A (Figure 5). This virtual complex $R_PF_iF_{3D/T}$ dissociates into post-termination complex R_P, F_i (RF_i) and F_{3D} (RF3·GDP) with compounded rate constant $k^{cmp}_{Pi3D/T}$.

In the second pathway **(B)** in Supplementary Figure S6B the post-termination complex R_PF_i (R_P·RF_i) binds F_{3T} (RF3·GTP) with compounded association rate constant k^{eff}_{Pi3T} in the formation of complex $R_PF_iF_{3T}$ (R_P·RF_i· RF3·GTP) from which dissociation F_i (RF_i) and F_{3D} (RF3·GDP) with compounded rate constant k_{Pi3} leads to ribosomal complex R_P.
<u>For spontaneous regeneration (reaction loop D)</u> of F_{3T} (RF3·GTP) from F_{3D} (RF3·GDP) in Supplementary Figure S6A, GDP dissociates from F_{3D} with rate constant q^s_{3D} , where superscript s signifies exchange in bulk solution, leading to free apo-RF3 (F_{3A}), which can either re-bind GDP (with rate constant k^s_{3D}) or bind GTP with rate constants k^s_{3D} in formation of free F_{3T} (RF3·GTP) in solution. The latter can re-form F_{3A} by GTP dissociation with rate constant q^s_{3T} or bind to post termination complex R_PF_i.

In the third pathway **(C)** F_i dissociates spontaneously from R_PF_i.
To demonstrate the identity of steady state properties of the schemes in Supplementary Figure S6B and Supplementary Figure S6A (Figure 5) we will first consider differential equations that

govern the time evolution of concentrations of ribosomal complexes depicted in Supplementary Figure S6A.

Ordinary differential equations (ODEs) and steady state equations for intracellular protein synthesis

The time evolutions of concentrations of ribosomal complexes in **Supplementary Figure S6A** are determined by the following set of ordinary differential equations (ODEs):

$$\frac{d}{dt}[R_E] = -k_E[R_E] + k_I[R_I]$$

$$\frac{d}{dt}[R_A] = -k_{Ai}[F_i][R_A] + q_{Ai}[R_A F_i] + k_E[R_E]$$

$$\frac{d}{dt}[R_A F_i] = k_{Ai}[F_i][R_A] - (k_{Hi} + q_{Ai})[R_A F_i]$$

$$\frac{d}{dt}[R_P F_i] = k_{Hi}[R_A F_i] - (q_{Pi} + k_{Pi3T}[F_{3T}] + k_{Pi3D}[F_{3D}])[R_P F_i] +$$
$$k_{Pi}[F_i][R_P] + q_{Pi3T}[R_P F_i F_{3T}] + q_{Pi3D}[R_P F_i F_{3D}]$$

$$\frac{d}{dt}[R_P F_i F_{3T}] = k_{Pi3T}[F_{3T}][R_P F_i] - (k_{Pi3} + q_{Pi3T})[R_P F_i F_{3T}]$$

$$\frac{d}{dt}[R_P F_i F_{3D}] = k_{Pi3D}[F_{3D}][R_P F_i] - (q_{3D} + q_{Pi3D})[R_P F_i F_{3D}] + k_{3D}[D][R_P F_i F_{3A}]$$

$$\frac{d}{dt}[R_P F_i F_{3A}] = q_{3D}[R_P F_i F_{3D}] - (k_{3D}[D] + k_{3T}[T])[R_P F_i F_{3A}] + q_{3T}[R_P F_i F_{3TA}]$$

$$\frac{d}{dt}[R_P F_i F_{3TA}] = k_{3T}[T][R_P F_i F_{3A}] - (q_{3T} + k_{3DPi})[R_P F_i F_{3TA}]$$

$$\frac{d}{dt}[R_P] = [R_P F_i]q_{Pi} + k_{Pi3}[R_P F_i F_{3T}] + k_{3DPi}[R_P F_i F_{3TA}] - k_{Pi}[F_i][R_P] - k_{P4}[F_4][R_P] \qquad \text{Eq. S1}$$

$$\frac{d}{dt}[R_P F_4] = k_{P4}[F_4][R_P] - k_{\mathrm{Rec}}[R_P F_4]$$

$$\frac{d}{dt}[R_I] = k_{\mathrm{Rec}}[R_P F_4] - k_I[R_I]$$

The time evolution of the GTP, apo- and GDP forms of free RF3 in solution are determined by:

$$\frac{d}{dt}[F_{3T}] = -k_{Pi3T}[F_{3T}][R_P F_i] + q_{Pi3T}[R_P F_i F_{3T}] + k^s_{3T}[T][F_{3A}] - q^s_{3T}[F_{3T}]$$

$$\frac{d}{dt}[F_{3D}] = k_{Pi3}[R_P F_i F_{3T}] + k_{3DPi}[R_P F_i F_{3TA}] - k_{Pi3D}[F_{3D}][R_P F_i] + q_{Pi3D}[R_P F_i F_{3D}]$$
$$-q^s_{3D}[F_{3D}] + k^s_{3D}[D][F_{3A}] \qquad \text{Eq. S2}$$

$$\frac{d}{dt}[F_{3A}] = q^s_{3D}[F_{3D}] - (k^s_{3D}[D] + k^s_{3T}[T])[F_{3A}] + q^s_{3T}[F_{3T}]$$

Here concentrations of free GTP and GDP in solution are denoted [T] and [D], respectively.

Steady state equations

The steady state concentrations of ribosomal complexes and free termination factors for the scheme in Supplementary Figure S6A are obtained by solving the algebraic equation system that arises when all time derivatives in Eqs. S1 and S2 are set to zero. We denote the steady state flow of protein molecules synthesized in a unit volume per time unit as j:

$$j = k_E [R_E]. \qquad \text{Eq. S3}$$

From this definition of *j* follows that:

$$[R_E] = \frac{j}{k_E}$$

$$[R_I] = \frac{j}{k_I}$$

$$[R_A] = \frac{j}{k_{Ai}^{eff}[F_i]}$$

$$[R_A F_i] = \frac{j}{k_{Hi}}$$

$$[R_P F_i F_{3T}] = \frac{k_{Pi3T}}{k_{Pi3} + q_{Pi3T}}[F_{3T}][R_P F_i] = \frac{k_{Pi3T}^{eff}}{k_{Pi3}}[F_{3T}][R_P F_i]$$

$$0 = k_{Hi}[R_A F_i] - (q_{Pi} + k_{Pi3T}[F_{3T}] + k_{Pi3D}[F_{3D}])[R_P F_i] +$$
$$k_{Pi}[F_i][R_P] + q_{Pi3T}[R_P F_i F_{3T}] + q_{Pi3D}[R_P F_i F_{3D}]$$

$$0 = k_{Pi3D}[F_{3D}][R_P F_i] - q_{Pi3D}[R_P F_i F_{3D}] - k_{3DPi}[R_P F_i F_{3TA}]$$
$$0 = q_{3D}[R_P F_i F_{3D}] - k_{3D}[D][R_P F_i F_{3A}] - k_{3DPi}[R_P F_i F_{3TA}]$$
$$0 = k_{3T}[T][R_P F_i F_{3A}] - (q_{3T} + k_{3DPi})[R_P F_i F_{3TA}]$$
$$j(1 + \frac{k_{Pi}[F_i]}{k_{P4}[F_4]}) = [R_P F_i] q_{Pi} + k_{Pi3}[R_P F_i F_{3T}] + k_{3DPi}[R_P F_i F_{3TA}] \qquad \text{Eq. S4}$$

$$[R_P] = \frac{j}{k_{P4}[F_4]}$$

$$[R_P F_4] = \frac{j}{k_{\text{Rec}}}$$

Here, we have introduced the effective (compounded) second order association rate constants k_{Ai}^{eff} and k_{Pi3T}^{eff} as:

$$k_{Ai}^{eff} = k_{Ai} / (1 + \frac{q_{Ai}}{k_{Hi}}), \qquad \text{Eq. S5}$$

$$k_{Pi3T}^{eff} = k_{Pi3T} / (1 + \frac{q_{Pi3T}}{k_{Pi3}}),$$

We note that these effective rate constants shown in Supplementary Figure S6B are both defined by a second order association rate constant for substrate binding multiplied by the

probability of product formation according to the general definition of the Michalis-Menten parameter k_{cat}/K_m.

Contraction of high resolution kinetics of Block A in Supplementary Figure S6A to low resolution kinetics of Supplementary Figure S6B.

To reduce the detailed kinetics of block A of Supplementary Figure S6A to block A of the contracted scheme in Supplementary Figure S6B with Michaelis-Menten parameters $k_{pi3D/T}^{eff}$ and k_{Pi3D}^{cmp} we first find the concentrations of ribosomal complexes in block A of Supplementary Figure S6A. Those are obtained from the following three equations in Eq. S4:

$$0 = k_{Pi3D}\left[F_{3D}\right]\left[R_P F_i\right] - q_{Pi3D}\left[R_P F_i F_{3D}\right] - k_{3DPi}\left[R_P F_i F_{3TA}\right]$$

$$0 = q_{3D}\left[R_P F_i F_{3D}\right] - k_{3D}\left[D\right]\left[R_P F_i F_{3A}\right] - k_{3DPi}\left[R_P F_i F_{3TA}\right]$$

$$0 = k_{3T}\left[T\right]\left[R_P F_i F_{3A}\right] - (q_{3T} + k_{3DPi})\left[R_P F_i F_{3TA}\right]$$

To get their solution we formally introduce $f = k_{3DPi}\left[R_P F_i F_{3TA}\right]$ and re-write the three equations as:

$$f = k_{Pi3D}\left[F_{3D}\right]\left[R_P F_i\right] - q_{Pi3D}\left[R_P F_i F_{3D}\right]$$

$$f = q_{3D}\left[R_P F_i F_{3D}\right] - k_{3D}\left[D\right]\left[R_P F_i F_{3A}\right] \qquad \text{Eq. S6}$$

$$\left[R_P F_i F_{3A}\right] = \frac{1}{k_{3T}\left[T\right]}(1 + \frac{q_{3T}}{k_{3DPi}}) f$$

Further algebraic manipulations lead to:

$$k_{Pi3D}\left[F_{3D}\right]\left[R_P F_i\right] = f(1 + q_{Pi3D}\frac{1}{q_{3D}}(1 + \frac{k_{3D}\left[D\right]}{k_{3T}\left[T\right]}(1 + \frac{q_{3T}}{k_{3DPi}})))$$

$$\left[R_P F_i F_{3D}\right] = \frac{f}{q_{3D}}(1 + \frac{k_{3D}\left[D\right]}{k_{3T}\left[T\right]}(1 + \frac{q_{3T}}{k_{3DPi}}))$$

$$\left[R_P F_i F_{3A}\right] = \frac{1}{k_{3T}\left[T\right]}(1 + \frac{q_{3T}}{k_{3DPi}}) f \qquad \text{Eq. S7}$$

$$\left[R_P F_i F_{3TA}\right] = \frac{f}{k_{3DPi}}$$

It then follows that:

$$f = k_{Pi3D}^{eff}\left[F_{3D}\right]\left[R_P F_i\right]$$

$$\left[R_P F_i F_{3D}\right] + \left[R_P F_i F_{3A}\right] + \left[R_P F_i F_{T3T}\right] = \frac{f}{k_{Pi3D}^{cmp}}, \qquad \text{Eq. S8}$$

where

$$k_{Pi3D/T}^{eff} = k_{Pi3D} / (1 + \frac{q_{Pi3D}}{q_{3D}}(1 + \frac{k_{3D}\left[D\right]}{k_{3T}\left[T\right]}(1 + \frac{q_{3T}}{k_{3DPi}}))), \qquad \text{Eq. S9}$$

and:

$$\frac{1}{k^{cmp}_{Pi3D/T}} = \frac{1}{q_{3D}}(1+\frac{k_{3D}[D]}{k_{3T}[T]}(1+\frac{q_{3T}}{k_{3DPi}})) + \frac{1}{k_{3T}[T]}(1+\frac{q_{3T}}{k_{3DPi}}) + \frac{1}{k_{3DPi}} \quad \text{Eq. S10}$$

In numeric simulations we assume [*D*]=0 and [*T*]=0.5 mM, so that $k^{eff}_{Pi3D/T}$ in Eq. S9 is [*T*]-independent. Moreover, q_{3D} is known to be very fast, so that at [*T*]=0.5 mM $k^{cmp}_{Pi3D/T}$ in Eq. S10 is mainly determined by k_{3DPi} value.

Clearly, the concentrations of $R_PF_iF_{3D}$, $R_PF_iF_{3A}$ and $R_PF_iF_{3TA}$ complexes can be expressed through the concentrations of R_PF_i and F_{3D} using Eq. S8 and Eq. S7.
We further notice that introducing virtual complex $R_PF_iF_{3D/T}$ with concentration

$$[R_PF_iF_{3D/T}] = [R_PF_iF_{3D}] + [R_PF_iF_{3A}] + [R_PF_iF_{3TA}], \quad \text{Eq. S11}$$

allows block A in Supplementary Figure S6A of the main text to be contracted to block A in Supplementary Figure S6B. $k^{eff}_{pi3D/T}$ in Eq. S9 is equal to the association rate constant k_{Pi3D} multiplied with the probability that ribosomal complex $R_PF_iF_{3D}$ in Supplementary Figure S6A leads to release of F_i .
The steady state concentrations of ribosomal complexes in Supplementary Figure S6B can then be expressed as:

$$[R_E] = \frac{j}{k_E}$$

$$[R_I] = \frac{j}{k_I}$$

$$[R_A] = \frac{j}{k^{eff}_{Ai}[F_i]}$$

$$[R_AF_i] = \frac{j}{k_{Hi}}$$

$$[R_PF_iF_{3T}] = \frac{k^{eff}_{Pi3T}}{k_{Pi3}}[F_{3T}][R_PF_i]$$

$$[R_PF_iF_{3D/T}] = [R_PF_iF_{3D}] + [R_PF_iF_{3A}] + [R_PF_iF_{3TA}] = \frac{k^{eff}_{Pi3D/T}[F_{3D}][R_PF_i]}{k^{cmp}_{Pi3D/T}}$$

$$[R_PF_i] = \frac{j}{(q_{Pi} + k^{eff}_{Pi3T}[F_{3T}] + k^{eff}_{Pi3D/T}[F_{3D}])}(1+\frac{k_{Pi}[F_i]}{k_{P4}[F_4]})$$

$$[R_P] = \frac{j}{k_{P4}[F_4]} \quad \text{Eq. S12}$$

$$[R_PF_4] = \frac{j}{k_{\mathrm{Re}c}}$$

Contraction of high resolution kinetics of Block D in Supplementary Figure S6A to low resolution kinetics of Block D in Supplementary Figure S6B.
It follows from Eq. S2 that the steady state concentrations of solution RF3 in the GTP, apo- and GDP forms in Supplementary Figure S6A satisfy the equation system:

$$0=-k_{Pi3T}\left[F_{3T}\right]\left[R_PF_i\right]+q_{Pi3T}\left[R_PF_iF_{3T}\right]+k_{3T}^{s}\left[T\right]\left[F_{3A}\right]-q_{3T}^{s}\left[F_{3T}\right]$$
$$0=k_{Pi3}\left[R_PF_iF_{3T}\right]+k_{3DPi}\left[R_PF_iF_{3TA}\right]-k_{Pi3D}\left[F_{3D}\right]\left[R_PF_i\right]+q_{Pi3D}\left[R_PF_iF_{3D}\right]$$
$$-q_{3D}^{s}\left[F_{3D}\right]+k_{3D}^{s}\left[D\right]\left[F_{3A}\right] \qquad \text{Eq. S13}$$
$$0=q_{3D}^{s}\left[F_{3D}\right]-(k_{3D}^{s}\left[D\right]+k_{3T}^{s}\left[T\right])\left[F_{3A}\right]+q_{3T}^{s}\left[F_{3T}\right]$$

Using Eqs S7 and S12 to express the steady state concentration of relevant ribosomal complexes through $\left[R_PF_i\right]$, Eq. S13 transforms into:

$$k_{Pi3T}^{eff}\left[F_{3T}\right]\left[R_PF_i\right]=k_{3T}^{s}\left[T\right]\left[F_{3A}\right]-q_{3T}^{s}\left[F_{3T}\right]$$
$$k_{Pi3T}^{eff}\left[F_{3T}\right]\left[R_PF_i\right]=q_{3D}^{s}\left[F_{3D}\right]-k_{3D}^{s}\left[D\right]\left[F_{3A}\right] \qquad \text{Eq. S14}$$
$$\left[F_{3A}\right]=\frac{q_{3D}^{s}\left[F_{3D}\right]+q_{3T}^{s}\left[F_{3T}\right]}{(k_{3D}^{s}\left[D\right]+k_{3T}^{s}\left[T\right])}$$

Substituting the F_{3A} concentration in the first two equations in Eq S14 one gets:

$$k_{Pi3T}^{eff}\left[F_{3T}\right]\left[R_PF_i\right]=q_{3D}^{s}\left[F_{3D}\right]\frac{k_{3T}^{s}\left[T\right]}{(k_{3D}^{s}\left[D\right]+k_{3T}^{s}\left[T\right])}-\frac{k_{3D}^{s}\left[D\right]}{(k_{3D}^{s}\left[D\right]+k_{3T}^{s}\left[T\right])}q_{3T}^{s}\left[F_{3T}\right], \qquad \text{Eq. S15}$$

which can be compacted to:

$$k_{Pi3T}^{eff}\left[R_PF_i\right]\left[F_{3T}\right]=k_{3F\_DT}^{cmp}\left[F_{3D}\right]-q_{3F\_DT}^{cmp}\left[F_{3T}\right] \qquad \text{Eq. S16}$$

where we introduced compounded rate constants as:

$$k_{3F\_DT}^{cmp}=q_{3D}^{s}\frac{k_{3T}^{s}\left[T\right]}{(k_{3D}^{s}\left[D\right]+k_{3T}^{s}\left[T\right])}\ , \qquad \text{Eq. S17}$$

and:

$$q_{3F\_DT}^{cmp}=q_{3T}^{s}\frac{k_{3D}^{s}\left[D\right]}{(k_{3D}^{s}\left[D\right]+k_{3T}^{s}\left[T\right])} \qquad \text{Eq. S18}$$

These relations explain the contraction of block D in Supplementary Figure S6A (Figure 5) to block D in Supplementary Figure S6B.
At intracellular concentrations of GTP and GDP the solution apo-form of RF3 exists but transiently, so that its presence can be neglected in further calculations. If, furthermore, free GTP concentration is much larger than free GDP concentration, Eq. S16 simplifies to:

$$k_{Pi3T}^{eff}\left[R_PF_i\right]\left[F_{3T}\right]=k_{3F\_DT}^{cmp}\left[F_{3D}\right] \qquad \text{Eq. S19}$$

Detailed (Figure 5, Supplementary Figure S6A) and reduced (Supplementary Figure S6B) schemes have identical steady state termination flows

The set of differential equations that governs the dynamics of the contracted scheme in Supplementary Figure S6B is:

$$\frac{d}{dt}[R_S] = -k_S[R_S] + k_{P4}[F_4][R_P]$$
$$\frac{d}{dt}[R_A] = -k_{Ai}^{eff}[F_i][R_A] + k_S[R_S]$$
$$\frac{d}{dt}[R_A F_i] = k_{Ai}^{eff}[F_i][R_A] - k_{Hi}[R_A F_i]$$
$$\frac{d}{dt}[R_P F_i] = k_{Hi}[R_A F_i] - (q_{Pi} + k_{Pi3T}^{eff}[F_{3T}] + k_{Pi3D/T}^{eff}[F_{3D}])[R_P F_i] + k_{Pi}[F_i][R_P]$$
$$\frac{d}{dt}[R_P F_i F_{3T}] = k_{Pi3T}^{eff}[F_{3T}][R_P F_i] - k_{Pi3}[R_P F_i F_{3T}]$$
$$\frac{d}{dt}[R_P F_i F_{3D/T}] = k_{Pi3D/T}^{eff}[F_{3D}][R_P F_i] - k_{Pi3D/T}^{cmp}[R_P F_i F_{3D/T}]$$
$$\frac{d}{dt}[R_P] = [R_P F_i] q_{Pi} + k_{Pi3}[R_P F_i F_{3T}] + k_{Pi3D/T}^{cmp}[R_P F_i F_{3D/T}]$$
$$-k_{Pi}[F_i][R_P] - k_{P4}[F_4][R_P]$$
$$\frac{d}{dt}[F_{3T}] = -k_{Pi3T}^{eff}[F_{3T}][R_P F_i] + k_{3F\_DT}^{cmp}[F_{3D}] - q_{3F\_DT}^{cmp}[F_{3T}]$$
$$\frac{d}{dt}[F_{3D}] = k_{Pi3}[R_P F_i F_{3T}] + q_{3F\_DT}^{cmp}[F_{3T}] + k_{Pi3D/T}^{cmp}[R_P F_i F_{3D/T}] \qquad \text{Eq. S20}$$
$$-k_{3F\_DT}^{cmp}[F_{3D}] - k_{Pi3D/T}^{eff}[F_{3D}][R_P F_i]$$

We note that a single virtual complex R_S with compounded life time $1/k_S$ in Supplementary Figure S6B formally replaces complexes R_E, R_I and R_PF_4 in Supplementary Figure S6A. Its concentration is obtained as the sum of concentrations R_E, R_I and R_PF_4:

$$[R_S] = [R_E] + [R_I] + [R_P F_4] = j\left(\frac{1}{k_E} + \frac{1}{k_I} + \frac{1}{k_{\mathrm{Rec}}}\right) = j\frac{1}{k_S}, \qquad \text{Eq.S21}$$

where

$$\frac{1}{k_S} = \frac{1}{k_E} + \frac{1}{k_I} + \frac{1}{k_{\mathrm{Rec}}} \qquad \text{Eq. S22}$$

It can be easily shown that the steady state concentrations of all complexes in Supplementary Figure S6A can be obtained from steady state concentrations of complexes in Supplementary Figure S6B using Eqs S7, S8, S12, S16 and S21. This proves that the kinetic schemes in Supplementary Figures S6A-S6B have identical steady state properties, despite that the relaxation trajectories to the steady state for the schemes in Supplementary Figures S6A-S6B may differ. This conclusion is important, since effective (see Eqs S5 and S9) and compounded (see Eqs. 10, 17 and 18) rate constants can be obtained experimentally (see Supplementary Table S1) much more easily that elemental rate constants in Figure 5 and Supplementary Figure S6A.

To actually find the steady state flows in the scheme in the Supplementary Figure S6B we numerically computed the time evolution of all concentrations using Eq. S20 with the initial conditions that RF3 is initially free and GDP bound and that all ribosomes are in elongation state R_E. The computation continues until all time derivatives in Eq. S20 are zero and the system is in the steady state. The steady state solution is completely defined by the rate constants and total concentrations of ribosomes and factors RF1 (F_1), RF2 (F_2,), RF3 (F_3) and the free concentration of RRF (F_4) in the scheme in Supplementary Figure S6B.

Steady state flows for termination on UAA codons read by both RF1 and RF2

We note that the scheme in Supplementary Figure S6B can be used to describe the steady state of ribosomal translation of mRNAs with UAG and UGA codons orthogonally read by RF1 and RF2, respectively. However, the case of translation of mRNAs with UAA stop codons read by both RF1 and RF2 requires a modified scheme shown in Supplementary Figure S7. In this scheme, RF1 and RF2 compete for pre-terminating ribosomes with UAA codons and RF3 acts on both RF1 and RF2 containing post-termination complexes. We also note that in Supplementary Figure S7 complexes R_P, R_A are targeted by both RF1 and RF2.

The differential equations that govern the time evolution of the concentrations of ribosomal complexes in Supplementary Figure S7 are:

$$\frac{d}{dt}[R_S] = -k_S[R_S] + k_{P4}[F_4][R_P]$$

$$\frac{d}{dt}[R_A] = -(k_{A1}^{eff}[F_1] + k_{A2}^{eff}[F_2])[R_A] + k_S[R_S]$$

$$\frac{d}{dt}[R_AF_1] = k_{A1}^{eff}[F_1][R_A] - k_{H1}[R_AF_1]$$

$$\frac{d}{dt}[R_PF_1] = k_{H1}[R_AF_1] - (q_{P1} + k_{P13T}^{eff}[F_{3T}] + k_{P13D/T}^{eff}[F_{3D}])[R_PF_1] + k_{P1}[F_1][R_P]$$

$$\frac{d}{dt}[R_PF_1F_{3T}] = k_{P13T}^{eff}[F_{3T}][R_PF_1] - k_{P13}[R_PF_1F_{3T}]$$

$$\frac{d}{dt}[R_PF_1F_{3D/T}] = k_{P13D/T}^{eff}[F_{3D}][R_PF_1] - k_{P13D/T}^{cmp}[R_PF_1F_{3D/T}]$$

$$\frac{d}{dt}[R_AF_2] = k_{A2}^{eff}[F_2][R_A] - k_{H2}[R_AF_2]$$

$$\frac{d}{dt}[R_PF_2] = k_{H2}[R_AF_2] - (q_{P2} + k_{P23T}^{eff}[F_{3T}] + k_{P23D/T}^{eff}[F_{3D}])[R_PF_2] + k_{P2}[F_2][R_P]$$

$$\frac{d}{dt}[R_PF_2F_{3T}] = k_{P23T}^{eff}[F_{3T}][R_PF_2] - k_{P23}[R_PF_2F_{3T}]$$

$$\frac{d}{dt}[R_PF_2F_{3D/T}] = k_{P23D/T}^{eff}[F_{3D}][R_PF_2] - k_{P23D/T}^{cmp}[R_PF_2F_{3D/T}]$$

$$\frac{d}{dt}[R_P] = [R_PF_1]q_{P1} + k_{P13}[R_PF_1F_{3T}] + k_{P13D/T}^{cmp}[R_PF_1F_{3D/T}] - k_{P1}[F_1][R_P]$$
$$+[R_PF_2]q_{P2} + k_{P23}[R_PF_2F_{3T}] + k_{P23D/T}^{cmp}[R_PF_2F_{3D/T}] - k_{P2}[F_2][R_P] - k_{P4}[c_4][R_P] \qquad \text{Eq. S23}$$

$$\frac{d}{dt}[F_{3T}] = -k_{P13T}^{eff}[F_{3T}][R_PF_1] - k_{P23T}^{eff}[F_{3T}][R_PF_2] + k_{3F\_DT}^{cmp}[F_{3D}] - q_{3F\_DT}^{cmp}[F_{3T}]$$

$$\frac{d}{dt}[F_{3D}] = k_{P13}[R_PF_1F_{3T}] + k_{P13D/T}^{cmp}[R_PF_1F_{3D/T}] - k_{P13D/T}^{eff}[F_{3D}][R_PF_1] +$$
$$k_{P23}[R_PF_2F_{3T}] + k_{P23D/T}^{cmp}[R_PF_2F_{3D/T}] - k_{P23D/T}^{eff}[c_{3D}][R_PF_2] - k_{3F\_DT}^{cmp}[F_{3D}] + q_{3F\_DT}^{cmp}[F_{3T}]$$

Numerical simulation of release factor dependent rates of termination and protein synthesis in living cells

To simulate translation in living cells where ribosomes translate UAG, UGA and UAA containing mRNAs, we assume for simplicity that concentrations of ribosomes translating UAA, UGA and UAG containing mRNAs remain constant as the system settles into the steady state. This is equivalent to the assumption that all ribosomes retain their mRNAs during the dynamic simulation and that the coupling between the synthesis of proteins by the ribosomes translating UAA, UGA and UAG mRNAs is due to their common use of intracellular release factors. The assumption is, in other words, that the connection between the dynamics (and, hence, the steady state concentrations) of ribosomal complexes with different types of mRNAs is determined by the concentrations of free RF1, RF2 and RF3.

The simulations are done using a combined equation set that include the set described in Eq. S24 for the UAA ribosomes plus two equations sets of Eq. S22 type for the UGA and UAG ribosomes. The combined equation set describes the joint evolution of 25 different ribosomal complex to their steady state. Relations between free to total concentrations of RF1 and RF2 follow directly from the law of matter conservation, i.e. from:

$$\begin{aligned}
&[F_{10}]=[F_1]+[R_AF_1]+[R_PF_1]+[RA_AF_1]+[RA_PF_1]+\\
&+[R_PF_1F_{3T}]+[R_PF_1F_{3D/T}]+[RA_PF_1F_{3T}]+[RA_PF_1F_{3D/T}]\\
&[F_{20}]=[F_2]+[R_AF_2]+[R_PF_2]+[RG_AF_2]+[RG_PF_2]+\\
&+[R_PF_2F_{3T}]+[R_PF_2F_{3D/T}]+[RG_PF_2F_{3T}]+[RG_PF_2F_{3D/T}]
\end{aligned} \qquad \text{Eq. S24}$$

Here, R, RA and RG are ribosomes translating UAA, UAG and UGA mRNAs, respectively. Free concentrations of RF1 ($[F_1]$) and RF2 ($[F_2]$) are calculated using Eq. S24 at each step of the numerical simulation from current concentrations of ribosomal complexes and known total concentrations of RF1 and RF2. The concentrations of free RF3 in the GTP and GDP forms are obtained from Eq. S25 at each step of numeric simulation using the current concentrations of ribosomal complexes:

$$\begin{aligned}
&\frac{d}{dt}[F_{3T}]=-k^{eff}_{P13T}[F_{3T}]([R_PF_1]+[RA_PF_1])-k^{eff}_{P23T}[F_{3T}]([R_PF_2]+[RG_PF_2])\\
&+k^{cmp}_{3F_DT}[F_{3D}]-q^{cmp}_{3F_DT}[F_{3T}]\\
&\frac{d}{dt}[F_{3D}]=k_{P13}[R_PF_1F_{3T}]+k^{cmp}_{P13D/T}[R_PF_1F_{3D/T}]-k^{eff}_{P13D/T}[F_{3D}][R_PF_1]+\\
&+k_{P23}[R_PF_2F_{3T}]+k^{cmp}_{P23D/T}[R_PF_2F_{3D/T}]-k^{eff}_{P23D/T}[c_{3D}][R_PF_2]+\\
&+k_{P13}[RA_PF_1F_{3T}]+k^{cmp}_{P13D/T}[RA_PF_1F_{3D/T}]-k^{eff}_{P13D/T}[F_{3D}][RA_PF_1]+\\
&+k_{P23}[RG_PF_2F_{3T}]+k^{cmp}_{P23D/T}[RG_PF_2F_{3D/T}]-k^{eff}_{P23D/T}[F_{3D}][RG_PF_2]\\
&-k^{cmp}_{3F_DT}[F_{3D}]+q^{cmp}_{3F_DT}[F_{3T}]
\end{aligned} \qquad \text{Eq. S25}$$

When running simulation we normally assume that free GTP concentration (about 0.5 mM in vivo) is much larger than free GDP concentration (assumed here to be near zero). This further implies that $q^{cmp}_{3F\_DT}\approx 0$ and $k^{cmp}_{3F\_DT}\approx q^{s}_{3D}$ meaning that if anything, our simulations overestimate

the contribution of spontaneous GTP exchange on RF3 in solution, and, hence, the contribution of pathway “B” operating effectively through pathway “D” (Figure 5) in termination.
The rate constants used for the numeric simulations shown in Figure 6 are summarized in Supplementary Table S1.
We note that there are three distinct phases in the class-I RF titrations without RF3 (Figure 6A). A first, starvation phase where the lifetime of ribosomal state R_A dominates the total termination time is observed for $[F_{Tot}] < 2$ µM, where $[F_{Tot}]$ is total concentration of class-I RFs in the cell (Figure 6A). Regarding RF2, the total termination time τ_{Term}^{UGA} on UGA and UAA codons is approximated by (see below, Eq. S47):

$$\tau_{Term}^{UGA} = \tau_{P2} \frac{[R_{Tot}]}{[F_{Tot2}]} - \tau_E \,, \qquad \text{Eq. S26}$$

where τ_{P2} = $1/q_{P2}$ is the mean time for RF2 dissociation from R_P·RF2 and τ_E is the elongation time of the ribosome (including the initiation and recycling times). Here $[R_{Tot}]/[F_{T2}] > \tau_E / \tau_{P2}$ and dominates τ_{Term}^{UGA} in Eq. S26, making it hypersensitive to $[R_{Tot}]/[F_{Tot2}]$ variation. Regarding RF1, inequalities $\tau_{P1} > \tau_{P2}$ (Supplementary Table S1) and $[F_{Tot2}] >> [F_{Tot1}]$ are always valid (Pavlov et al., 1999; Baggett et al, PLOS, 2017), so that starvation phase for RF1 is more severe, i.e. $\tau_{Term}^{UAG} >> \tau_{Term}^{UGA}$.
A second, factor dissociation phase is observed at $[F_{Tot}]$ values just above 2 µM where free concentration of RF2 is so large that τ_{Term}^{UGA} is dominated by meant time $1/q_{p2}$ of its spontaneous dissociation $\tau_{Term}^{UGA} \approx \tau_{Term}^{UAA} \approx 1/q_{P2} = 1\,s$ (Figure 5).
A third, ribosomal recycling inhibition phase becomes apparent at high $[F_{Tot}]$ values (Figure 6A, red). Here, when $[F_{Tot}]$ increases then τ_{Term}^{UGA} increases gradually due to increased rebinding of RF2 back to post-terminated ribosomes with empty A-site (R_P) in competition with RRF (F_4) and EF-G thereby inhibiting ribosome splitting into subunits and delaying initiation complex (R_I) formation.

Analytical solution for steady state concentrations of the scheme in Supplementary Figure S6B

It is instructive to obtain analytic expressions for the time of termination for the scheme in Supplementary Figure S6B, and, hence also for that in Figure 5. To this end we first use that the concentrations of ribosomal complexes expressed through the flow j in Eq. S12 must sum up to the total ribosome concentration denoted here as $[R_0]$, i.e.:

$$[R_0] = ([R_P] + [R_P F_4] + [R_I] + [R_E]) + [R_A] + [R_A F_i] + \{[R_P F_i F_{3T}] + [R_P F_i F_{3D/T}] + [R_P F_i]\} =$$

$$= \left(\frac{j}{k_{P4}[F_4]} + \frac{j}{k_I} + \frac{j}{k_E} + \frac{j}{k_{\mathrm{Rec}}}\right) + \frac{j}{k_{Ai}^{eff}[F_i]} + \frac{j}{k_{Hi}} + \qquad \text{Eq. S27}$$

$$\left\{1 + \frac{k_{Pi3T}^{eff}}{k_{Pi3}}[F_{3T}] + \frac{k_{Pi3D/T}^{eff}}{k_{Pi3D/T}^{cmp}}[F_{3D}]\right\} \frac{j}{(q_{Pi} + k_{Pi3T}^{eff}[F_{3T}] + k_{Pi3D/T}^{eff}[F_{3D}])} (1 + \frac{k_{Pi}[F_i]}{k_{P4}[F_4]})$$

Eq. S27 relates flow “j” and rate constants in Supplementary Figure S6B, which, in a more compact form, can be written as:

$$\tau_R = \frac{[R_0]}{j} = \tau_{\min} + \frac{1}{k_{Ai}^{eff}[F_i]} + \frac{1}{k_{Hi}} + \tau_{RFi}(1+\frac{k_{Pi}[F_i]}{k_{P4}[F_4]}) \quad \text{Eq. S28}$$

Here, τ_R is the time it takes for a ribosome to go through one cycle of synthesis of a 300 aa protein including all the steps depicted in Supplementary Figure S6B; we have also denoted the sum of times from RRF(F_4) binding to R_P, recycling, initiation and elongation as $\tau_{\min}$:

$$\tau_{\min} = \frac{1}{k_{P4}[c_4]} + \frac{1}{k_{\mathrm{Rec}}} + \frac{1}{k_I} + \frac{1}{k_E} \quad \text{Eq. S29}$$

Further, τ_{PRFi} in Eq. S28 is the sum of times the ribosome spends in post- release complexes $[R_P F_i]$ $[R_P F_i F_{3T}]$ and $[R_P F_i F_{3D/T}]$ (the last two contain RF3) under conditions of “infinite” RRF (F_4) concentration, i.e.:

$$\tau_{PRFi} = \left\{1 + \frac{k_{Pi3T}^{eff}}{k_{Pi3}}[F_{3T}] + \frac{k_{Pi3D/T}^{eff}}{k_{Pi3D/T}^{cmp}}[F_{3D}]\right\}\frac{j}{(q_{Pi} + k_{Pi3T}^{eff}[F_{3T}] + k_{Pi3D/T}^{eff}[F_{3D}])} \quad \text{Eq. S30}$$

We note that τ_{RFi} variations are only due to the variations in the concentrations of free RF3•GDP ($[F_{3T}]$) and RF3•GDP ($[F_{3D}]$). Using Eq. S28 to get an expression for τ_R the free concentrations of RFi, RF3•GDP and RF3•GTP must somehow be obtained. To find $[F_i]$ we introduce the fraction x of total RFi (F_{i0}) concentration free in solution:

$$x = \frac{[F_i]}{[F_{i0}]} \quad \text{Eq. S31}$$

We also introduce the notations:

$$\tau_{w0} = \frac{1}{k_{Ai}^{eff}[F_{i0}]}, \quad \text{Eq. S32}$$

and:

$$\alpha = \frac{k_{Pi}[F_{i0}]}{k_{P4}[F_4]} \quad \text{Eq. S33}$$

Then Eq. S28 transforms to:

$$\tau_R = \frac{[R_0]}{j} = \tau_{\min} + \frac{\tau_{w0}}{x} + \frac{1}{k_{Hi}} + \tau_{PRFi}(1+\alpha \cdot x) \quad \text{Eq. S34}$$

We note that the concentration of ribosome bound RF_i can be expressed through the flow j as (see Eq. S12):

$$[R_A F_i] + [R_P F_i] + [R_P F_i F_{3T}] + [R_P F_i F_{3D/T}] = j(\frac{1}{k_{Hi}} + \tau_{PRFi}(1+\alpha \cdot x)) \quad \text{Eq. S35}$$

Then the concentration of free F_i is:

$$[F_i] = x[F_{i0}] = [F_{i0}] - ([R_A F_i] + [R_P F_i] + [R_P F_i F_{3T}] + [R_P F_i F_{3D/T}])$$

$$= [F_{i0}] - j(\frac{1}{k_{Hi}} + \tau_{PRFi}(1 + \alpha \cdot x)) \qquad \text{Eq. S36}$$

Combining Eq. S36 with Eq. S34 one obtains the following equation for x:

$$x = 1 - \frac{j}{[F_{i0}]}(\frac{1}{k_{Hi}} + \tau_{PRFi}(1 + \alpha \cdot x)) =$$

$$1 - y_0(\frac{1}{k_{Hi}} + \tau_{PRFi}(1 + \alpha \cdot x)) / \left\{ \tau_{\min} + \frac{\tau_{w0}}{x} + \frac{1}{k_{Hi}} + \tau_{PRFi}(1 + \alpha \cdot x) \right\} \qquad \text{Eq. S37}$$

Here, y_0 is the ratio between total ribosome and total RF_i concentrations:

$$y_0 = \frac{[R_0]}{[F_{i0}]} \qquad \text{Eq. S38}$$

Further algebraic manipulations transform Eq. S37 into a cubic equation for x:

$$\frac{\alpha \tau_{PRFi}}{\tau_{\min} + \tau_{RFi}} \cdot x^3 + x^2 (1 + \frac{\alpha \tau_{PRFi}(y_0 - 1)}{\tau_{\min} + \tau_{RFi}}) + \left\{ \frac{y_0 \tau_{RFi} + \tau_{w0}}{\tau_{RFi} + \tau_{\min}} - 1 \right\} x - \frac{\tau_{w0}}{\tau_{RFi} + \tau_{\min}} = 0, \qquad \text{Eq. S39}$$

where

$$\tau_{RFi} = \frac{1}{k_{Hi}} + \tau_{PRFi} \qquad \text{Eq. S40}$$

Knowing x one obtains the total termination time as:

$$\tau_{Term} = \frac{\tau_{w0}}{x} + \frac{1}{k_{Hi}} + \tau_{PRFi}(1 + \alpha \cdot x) \qquad \text{Eq. S41}$$

In addition to the termination time one obtains the time, τ_w, the pre-termination ribosome waits for RF_i binding with the empty A site containing a stop codon as:

$$\tau_w = \frac{\tau_{w0}}{x} \qquad \text{Eq. S42}$$

The term $(1 + \alpha \cdot x)$ in Eq. S42 gives an average number of RF_i re-bindings to the post-termination complex R_P before F_4 (RRF) bind productively to it and, together with EF-G (not shown on the schemes), irreversibly disassemble post-termination ribosome R_P it into ribosomal subunits (Borg et al, 2016).

We also note that when the steady state concentrations of ribosomal complexes and release factors are found numerically we can use Eq. S3 to calculate flow j and then find the time the factor is bound to the ribosome, $1/k_{Hi} + \tau_{RFi}(1 + \alpha \cdot x)$, from Eq. S35 . Further, taking also into account that

$$\tau_w = \frac{j}{k_{Ai}^{eff}[F_i]}, \qquad \text{Eq. S43}$$

the waiting time for RF_i binding in the case of numerical solution can be calculated and Eq. S41 can then be used to obtain the termination time τ_{Term}.

We note also that Eq. S39 simplifies to quadratic form when re-binding of RF_i to post-termination ribosomes is negligible. This occurs when the concentration of RRF is high in comparison with $[F_{i0}]$, a condition normally fulfilled at physiological concentrations of RRF and F_{i0}. Then $\alpha \approx 0$ and one obtains:

$$x^2 + x\left[\frac{\tau_{w0} + y_0\tau_{RFi}}{\tau_{\min} + \tau_{RFi}} - 1\right] - \frac{\tau_{w0}}{\tau_{\min} + \tau_{RFi}} = 0 \qquad \text{Eq. S44}$$

It follows from Eq. S44 that when $(y_0 - 1)\tau_{RFi} >> \tau_{\min}$ (i.e. when the ribosomes are in large excess over class one release factors and the dwell time, τ_{RFi}, of release factor on the ribosome is long) the fraction of free release factor can be approximated as:

$$x \approx \frac{\tau_{w0}}{(\tau_{w0} - \tau_{\min} + (y_0 - 1)\tau_{RFi})} \qquad \text{Eq. S45}$$

Substituting this approximation for x into Eq. 41 (and recalling also that $\alpha \approx 0$) one obtains:

$$\tau_{Term} = \frac{\tau_{w0}}{x} + \tau_{RFi} \approx \tau_{w0} + y_0\tau_{RFi} - \tau_{\min} = \frac{[R_0]}{[F_{i0}]}\left\{\frac{1}{k_{Ai}^{eff}[R_0]} + \tau_{RFi}\right\} - \tau_{\min} \qquad \text{Eq. S46}$$

We note that termination time τ_{Term} in the approximate Eq. S46 is totally dominated by the time, τ_w, the pre-termination ribosome waits for RF_i binding. Assuming further that the total concentration of ribosomes, $[R_0]$, is high enough to neglect $1/k_{Ai}^{eff}[R_0]$ in comparison with τ_{RFi} Eq. S46 simplifies to

$$\tau_{Term} \approx \frac{[R_0]}{[F_{i0}]}\tau_{RFi} - \tau_{\min} \qquad \text{Eq. S47}$$

Interestingly, and somewhat contra-intuitively, Eq. 47 shows that under these conditions the termination time τ_{Term} does not actually depend on the effective association rate k_{Ai}^{eff} of RF_i to pre-terminating ribosome.

The total ribosome cycle time, τ_R, can be approximated as:

$$\tau_R = \tau_{Term} + \tau_{\min} = \frac{[R_0]}{[F_{i0}]}\left\{\frac{1}{k_{Ai}^{eff}[R_0]} + \tau_{RFi}\right\} \approx \frac{[R_0]}{[F_{i0}]}\tau_{RFi} \qquad \text{Eq. S48}$$

It shows that for long dwell times τ_{RFi} of F_i on the ribosome, the time of the ribosome cycle is inversely proportional to the total concentration of RF_i in the cell.

Uniqueness of the steady state solutions

We note that the existence and the uniqueness of the steady state requires that Eq. S44 or Eq. S39 has one and only one physically meaningful root, i.e. the root in the interval between 0 and 1 (see Eq. S31 for "x" definition). That it is the case can be easily proven for the only positive root of Eq. S44. Indeed, the negativity of the free term of Eq. S44 implies that its two roots have different signs. To confirm that the positive root is smaller than 1 we can make the substitution x=y+1 in Eq. S44 resulting in the following equation for y:

$$y^2 + y(1+\frac{\tau_{w0} + y_0\tau_{RFi}}{\tau_{\min} + \tau_{RFi}}) + \frac{y_0\tau_{RFi}}{\tau_{\min} + \tau_{RFi}} = 0 \qquad \text{Eq. S49}$$

The positivity of the free term in Eq. S49 implies that the y-roots have the same sign and, moreover, from the positivity of the y-coefficient it follows that the both roots are negative. It then follows that both x-roots of Eq. S44 (obtained by +1 shift of y-roots) are smaller than 1 and, hence, the only positive x-root of Eq. S44 is the unique solution of Eq. S44 with a physical meaning.

The same conclusion holds for cubic Eq. S39. To see this we first re-write it as:

$$x^3 + x^2(\frac{\tau_{\min} + \tau_{RFi}}{\alpha\tau_{PRFi}} + (y_0 - 1)) + \left\{\frac{(y_0 - 1)\tau_{RFi} + \tau_{w0} - \tau_{\min}}{\alpha\tau_{PRFi}}\right\}x - \frac{\tau_{w0}}{\alpha\tau_{PRFi}} = 0 \qquad \text{Eq. S50}$$

Since the free term is negative the equation can have: (i) one positive and two negative real x-roots (ii) one positive real and two complex conjugated x-roots or (iii) three real positive x-roots. The last possibility is certainly excluded when $y_0 > 1$ since for $y_0 > 1$ the x^2 coefficient in Eq. S50 is always positive. Making the substitution x = 1 + y one obtains:

$$y^3 + y^2(\frac{\tau_{\min} + \tau_{RFi}}{\alpha\tau_{PRFi}} + 2 + y_0) + \left\{\frac{(y_0 + 1)\tau_{RFi} + \tau_{w0} + \tau_{\min}}{\alpha\tau_{PRFi}} + 1 + 2y_0\right\}y + \left[\frac{y_0\tau_{RFi}}{\alpha\tau_{PRFi}} + y_0\right] = 0 \qquad \text{Eq. S51}$$

The strict positivity of the free term and y^2 coefficient implies that the case (i) of x-roots corresponds to the case (i) of three negative real y-roots meaning that the only positive x-root is between 1 and zero and hence is the only physically feasible solution; the case (ii) of x-roots corresponds to one negative real and two complex conjugated y-roots, and, hence the only positive x-root is again between 1 and zero; finally, the case (iii) of x-roots leads to case (iii) of one negative and two positive real y- roots implies that only one positive x-root in between 1 and zero meaning that in all case Eq. S50 has one and only one real x-root between 1 and 0 establishing the uniqueness of the steady state in termination.

Analytical solution of Eq. S25 in the presence of RF3

The cubic and quadratic equations for “x” discussed above are easily solved in the absence of RF3 (F_3) when $\tau_{PRFi} = 1/q_{Pi}$ (q_{Pi} is the rate constant for spontaneous dissociation of RF_i from R_P•RF_i). However, in the RF3 presence τ_{PRFi} in Eq. S39 depends on concentrations of free RF3•GDP (F_{3D}) and RF3•GTP (F_{3T}); those need to be known as functions of “x” to actually solve the equation. This can be accomplished with a relative ease for the case when $q_{3F\_DT}^{cmp}$ (see Eq.S18) is very small and can be neglected so that Eq. S19 is valid. We note that this corresponds, in effect, to an overestimation of the contribution of the GDP to GTP exchange in solution to RF3 function. To obtain the equation to calculate free F_{3D} and F_{3T} concentrations when RF3 is present at total concentration $\left[F_{30}\right]$ and when $q_{3F\_DT}^{cmp}$ is set to zero we use the following relations (see Eq. S12 and Eq. S19):

$$[R_P F_i F_{3T}] = \frac{k_{Pi3T}^{eff}}{k_{Pi3}}[F_{3T}][R_P F_i]$$

$$[R_P F_i F_{3D/T}] = \frac{k_{Pi3D/T}^{eff}}{k_{Pi3D/T}^{cmp}}[F_{3D}][R_P F_i] \qquad \text{Eq. S52}$$

$$[F_{3D}] = \frac{k_{Pi3T}^{eff}}{k_{3F\_DT}^{cmp}}[F_{3T}][R_P F_i]$$

$$[F_{3T}] = [F_{3D}]\frac{k_{3F\_DT}^{cmp}}{k_{Pi3T}^{eff}}\frac{1}{[R_P F_i]}$$

The conservation law for the scheme in Supplementary Figure S6B implies that:

$$[F_{30}] = [F_{3T}] + [F_{3D}] + [R_P F_i F_{3T}] + [R_P F_i F_{3D/T}] =$$
$$= [F_{3D}]\frac{k_{3F\_DT}^{cmp}}{k_{Pi3T}^{eff}[R_P F_i]} + [F_{3D}] + \frac{k_{3F\_DT}^{cmp}}{k_{Pi3}}[F_{3D}] + \frac{k_{Pi3D/T}^{eff}}{k_{Pi3D/T}^{cmp}}[F_{3D}][R_P F_i] \qquad \text{Eq. S53}$$

This reduces to:

$$\frac{1}{z_D} = (1 + \frac{k_{3F\_DT}^{cmp}}{k_{Pi3}}) + \frac{k_{3F\_DT}^{cmp}}{k_{Pi3T}^{eff}[R_P F_i]} + \frac{k_{Pi3D/T}^{eff}}{k_{Pi3D/T}^{cmp}}[R_P F_i], \qquad \text{Eq. S54}$$

where we have denoted the fraction of free RF3·GDP as z_D:

$$z_D = \frac{[F_{3D}]}{[F_{30}]} \qquad \text{Eq. S55}$$

We note that Eq. S54 implies that the fraction of total RF3 in the F_{3D} form (i.e. in solution and containing GDP) is completely determined by rate constant of the scheme in Supplementary Figure S6B and the concentration of the post termination complex R_PF_i. Further, using Eq. S12 again, we can relate the concentration $[F_{i0}](1-x)$ of ribosome bound F_i to the concentration of R_PF_i complex:

$$[F_{i0}](1-x) = [R_A F_i] + [R_P F_i] + [R_P F_i F_{3T}] + [R_P F_i F_{3D/T}] =$$
$$= \frac{1}{k_{Hi}(x)}[R_P F_i](q_{Pi} + \frac{k_{3F\_DT}^{cmp}}{[R_P F_i]}[F_{3D}] + k_{Pi3D/T}^{eff}[F_{3D}]) + [R_P F_i] \qquad \text{Eq. S56}$$
$$+ \frac{1}{k_{Pi3}}\frac{k_{3F\_DT}^{cmp}}{[R_P F_i]}[F_{3D}][R_P F_i] + \frac{k_{Pi3D/T}^{eff}}{k_{Pi3D/T}^{cmp}}[F_{3D}][R_P F_i]$$

Here we introduced the notation:

$$k_{Hi}(x) = k_{Hi}(1+\alpha x) \qquad \text{Eq. S57}$$

From the last relation in Eq. S52 it follows that Eq. S56 can be re-written as:

$$[R_P F_i] = \left\{\frac{[F_{x0}]}{[F_{30}] z_D}(1-x) - k_{3F\_DT}^{cmp}\left[\frac{1}{k_{Hi}(x)} + \frac{1}{k_{Pi3}}\right]\right\} /$$
$$\left\{\left[1 + \frac{q_{Pi}}{k_{Hi}(x)}\right]\frac{1}{[F_{30}] z_D} + k_{Pi3D/T}^{eff}\left[\frac{1}{k_{Hi}(x)} + \frac{1}{k_{Pi3D/T}^{cmp}}\right]\right\} \qquad \text{Eq. S58}$$

We can then substitute Eq. S58 into Eq. S54 to obtain the following cubic equation for z_D:

$$z_D^3\left\{\frac{k_{Pi3D/T}^{eff}}{k_{Pi3D/T}^{cmp}}ab^2+d^2\frac{k_{3F\_DT}^{cmp}}{k_{Pi3T}^{eff}a}-bd(1+\frac{k_{3F\_DT}^{cmp}}{k_{Pi3}})\right\}+$$

$$+z_D^2\left\{(1+\frac{k_{3F\_DT}^{cmp}}{k_{Pi3}})(d-b)+bd+2\frac{k_{3F\_DT}^{cmp}}{k_{Pi3T}^{eff}a}d-2\frac{k_{Pi3D/T}^{eff}}{k_{Pi3D/T}^{cmp}}ab\right\}+ \qquad \text{Eq. S59}$$

$$+z_D\left\{1+\frac{k_{3F\_DT}^{cmp}}{k_{Pi3}}+\frac{k_{3F\_DT}^{cmp}}{k_{Pi3T}^{eff}a}+\frac{k_{Pi3D/T}^{eff}}{k_{Pi3D/T}^{cmp}}a-d+b\right\}-1=0$$

Here, for the sake of compactness of coefficients of the cubic equation Eq. S59, we introduced the following notations:

$$a=\left[F_{i0}\right](1-x)/(1+\frac{q_{Pi}}{k_{Hi}(x)})$$

$$b=\frac{k_{3F\_DT}^{cmp}\left[F_{30}\right]}{\left[F_{i0}\right](1-x)}\left[\frac{1}{k_{Hi}(x)}+\frac{1}{k_{Pi3}}\right] \qquad \text{Eq. S60}$$

$$d=k_{Pi3D/T}^{eff}\left[F_{30}\right]\left[\frac{1}{k_{Hi}(x)}+\frac{1}{k_{Pi3D/T}^{cmp}}\right]/(1+\frac{q_{Pi}}{k_{Hi}(x)})$$

It is seen that coefficients a, b and d in Eq. S60 are functions of x, rate constants of the kinetic scheme in Supplementary Figure S6B and total concentrations of RFi and RF3.
Both x and z_D can now be found by solving the system of two cubic equations S39 and S59 by e.g. sequential iterations using the x value obtained from Eq. S39 to calculate the coefficients of the cubic equation for z_D (Eq. S59). Next, the so computed z_D is used in Eq. S57 to find $\left[R_PF_i\right]$, and then the F_{3T} concentration is found from the last expression in Eq. S52. Having the F_{3T} and F_{3D} concentrations we re-calculate τ_{PRFi} using Eq. S30 and this τ_{PRFi} is then used in Eq. S39 in the next iteration to find a new x. This iterative procedure of solving non-linear equation systems normally converges very fast to the steady state values of x and z_D.
We have checked that the semi-analytic way described above to find x and z_D for the scheme in Supplementary Figure S6B gives the same values of termination times as does the direct numerical method of propagating the system to the steady state using the differential equation system Eq. S12.

Contribution of GDP to GTP exchange on free RF3 in solution to rate of termination in the living cell

Let us introduce the fraction "v" of RF3 (F_3)-dependent F_i dissociation flow that proceeds through the action of RF3:GTP (F_{3T}) formed through GDP to GTP exchanged in solution. This fraction is given by:

$$v=\frac{\left[R_PF_iF_{3T}\right]k_{Pi3}}{\left[R_PF_iF_{3T}\right]k_{Pi3}+\left[R_PF_iF_{3D/T}\right]k_{Pi3D/T}^{cmp}} \qquad \text{Eq. S61}$$

Using relations in Eq. S52 it follows that:

$$v = \frac{1}{1 + k_{Pi3D/T}^{eff} \left[R_P F_i \right] / k_{3F\_DT}^{cmp}},$$ Eq. S62

Further, since at low total RF3 concentration in the cell a considerable fraction of class-I release factors will be ribosome bond we will have $k_{Pi3D/T}^{eff} \left[R_P F_i \right] / k_{3F\_DT}^{cmp} >> 1$ and, hence, the fraction v is very small. Indeed, the modeling shows that at standard in vivo concentrations of release factor v is smaller than 3% indicating a negligible contribution of solution GDP to GTP exchange on RF3 to the bacterial termination.

References

Borg A, Pavlov M, Ehrenberg M (2016) Complete kinetic mechanism for recycling of the bacterial ribosome. RNA 22: 10-21

Indrisiunaite G, Pavlov MY, Heurgue-Hamard V, Ehrenberg M (2015) On the pH dependence of class-1 RF-dependent termination of mRNA translation. J Mol Biol 427: 1848-1860

Pavlov MY, Freistroffer DV, Heurgue-Hamard V, Buckingham RH, Ehrenberg M (1997) Release factor RF3 abolishes competition between release factor RF1 and ribosome recycling factor (RRF) for a ribosome binding site. J Mol Biol 273: 389-401

Peske F, Kuhlenkoetter S, Rodnina MV, Wintermeyer W (2014) Timing of GTP binding and hydrolysis by translation termination factor RF3. Nucleic Acids Res 42: 1812-1820

Zavialov AV, Buckingham RH, Ehrenberg M (2001) A posttermination ribosomal complex is the guanine nucleotide exchange factor for peptide release factor RF3. Cell 107: 115-124

Zavialov AV, Mora L, Buckingham RH, Ehrenberg M (2002) Release of peptide promoted by the GGQ motif of class 1 release factors regulates the GTPase activity of RF3. Mol Cell 10: 789-798

Zorzet A, Pavlov MY, Nilsson AI, Ehrenberg M, Andersson DI (2010) Error-prone initiation factor 2 mutations reduce the fitness cost of antibiotic resistance. Mol Microbiol 75: 1299-1313
